# Combinatorial effects of gene dosage, polygenic background and environment on complex traits

**DOI:** 10.64898/2026.04.30.26352063

**Published:** 2026-05-01

**Authors:** Molly F. Sacks, Marieke Klein, Tim B. Bigdeli, Mart Kals, Matthew T. Oetjens, Florian Bénitière, Jacquelyn Johnson, Adam Maihofer, Margit Nõukas, Michael Francis, Bryan Gorman, Iskander Said, Giulio Genovese, Georgios Voloudakis, Kyriacos Markianos, Murray Stein, Joel Gelernter, David H. Ledbetter, Caroline M. Nievergelt, Christa Lese Martin, Vincent-Raphaël Bourque, Omar Shanta, Jeffrey R. MacDonald, Bhooma Thiruvahindrapuram, Mamad Ahangari, Anjali Srinivasan, James Guevara, Jessica H. Hall, Josephine E. Haddon, Claudia Vingerhoets, David Linden, Mieke M. van Haelst, Marianne B.M. van den Bree, Carrie E. Bearden, Raquel E. Gur, T. Blaine Crowley, Daniel E. McGinn, Beverly S. Emanuel, Elaine H. Zackai, Ann Swillen, Thérèse van Amelsvoort, Jacob Vorstman, Anne S. Bassett, Donna M. McDonald-McGinn, Panos Roussos, Mihaela Aslan, Philip D. Harvey, Million Veteran Program, Estonian Biobank Research Team, Genes to Mental Health Network, Sébastien Jacquemont, Saiju Pyarajan, Kelli Lehto, Peter M. Visscher, Jonathan Sebat

**Affiliations:** 1). Bioinformatics and Systems Biology Graduate Program, University of California San Diego, La Jolla, CA, USA; 2). Department of Psychiatry and Department of Cellular & Molecular Medicine, University of California San Diego, La Jolla, CA, USA; 3). Department of Medical Neuroscience, Donders Institute for Brain, Cognition and Behaviour, Radboud University Medical Center, Nijmegen, The Netherlands; 4). VA New York Harbor Healthcare System, New York, NY, USA; 5). Institute for Genomics in Health (IGH), SUNY Downstate Health Sciences University, New York, NY, USA; 6). Department of Psychiatry and Behavioral Sciences, SUNY Downstate Health Sciences University, New York, NY, USA; 7). Department of Epidemiology and Biostatistics, School of Public Health, SUNY Downstate Health Sciences University, New York, NY, USA; 8). Estonian Genome Centre, Institute of Genomics, University of Tartu, Tartu, Estonia; 9). Department of Developmental Medicine, Geisinger College of Health Sciences, Danville, PA, USA; 10). CHU Sainte-Justine Research Centre, Université de Montréal, Canada; 11). Research Service, Veterans Affairs San Diego Healthcare System, San Diego, CA, USA; 12). Center for Data and Computational Sciences (C-DACS), VA Boston Healthcare System, Boston, MA, USA; 13). Booz Allen Hamilton, McLean, VA, USA; 14). Stanley Center, Broad Institute of MIT and Harvard, Cambridge, MA, USA; 15). Program in Medical and Population Genetics, Broad Institute of MIT and Harvard, Cambridge, MA, USA; 16). Department of Genetics, Harvard Medical School, Boston, MA, USA; 17). Center for Disease Neurogenomics, Icahn School of Medicine at Mount Sinai, New York, NY, USA; 18). Friedman Brain Institute, Icahn School of Medicine at Mount Sinai, New York, NY, USA; 19). Department of Psychiatry, Icahn School of Medicine at Mount Sinai, New York, NY, USA; 20). Department of Genetics and Genomic Sciences, Icahn School of Medicine at Mount Sinai, New York, NY, USA; 21). Center for Precision Medicine and Translational Therapeutics, James J. Peters VA Medical Center, Bronx, NY, USA; 22). Mental Illness Research, Education and Clinical Center, James J. Peters VA Medical Center, Bronx, NY, USA; 23). Herbert Wertheim School of Public Health and Human Longevity Science, University of California San Diego, La Jolla, CA, USA; 24). Department of Psychiatry, Yale University School of Medicine, New Haven, CT, USA; 25). Department of Genetics, Yale University School of Medicine, New Haven, CT, USA; 26). Department of Psychiatry, VA Connecticut Healthcare System, West Haven, CT, USA; 27). Florida Institute for Pediatric Rare Diseases, Tallahassee, FL, USA; 28). Florida State University College of Medicine, Tallahassee, FL, USA; 29). Division of Child Psychiatry, McGill University, Montréal, Canada; 30). The Centre for Applied Genomics, The Hospital for Sick Children, Toronto, Ontario, Canada; 31). Program in Genetics and Genome Biology, The Hospital for Sick Children, Toronto, Ontario, Canada; 32). Division of Psychological Medicine and Clinical Neurosciences, School of Medicine, Cardiff University, Cardiff, UK; 33). Cardiff University Brain Research Imaging Centre (CUBRIC), Cardiff University, Cardiff, UK; 34). Division of Psychological Medicine and Clinical Neurosciences, Centre for Neuropsychiatric Genetics and Genomics, Cardiff University, Cardiff, UK; 35). Neuroscience and Mental Health Innovation Institute, Cardiff University, Cardiff, UK; 36). School for Mental Health and Neuroscience, Department of Psychiatry and Neuropsychology, Faculty of Health, Medicine and Life Sciences, Maastricht University, Maastricht, the Netherlands; 37). Advisium’s Heeren Loo Zorggroep, Amersfoort, The Netherlands; 38). Amsterdam UMC, University of Amsterdam, Department of Human Genetics, Meibergdreef, Amsterdam, The Netherlands; 39). Amsterdam Reproduction and Development Research Institute, Amsterdam, The Netherlands; 40). Amsterdam UMC, University of Amsterdam, Emma Center for Personalized Medicine, Meibergdreef, Amsterdam, The Netherlands; 41). Centre for Neuropsychiatric Genetics and Genomics, Cardiff University, UK; 42). Neuroscience and Mental Health Innovation Institute, Cardiff University, UK; 43). Department of Psychiatry and Biobehavioral Sciences, Semel Institute for Neuroscience and Human Behavior, University of California, Los Angeles, CA, USA; 44). Department of Psychology, University of California, Los Angeles, CA, USA; 45). Department of Psychiatry, University of Pennsylvania, Philadelphia, PA, USA; 46). Children’s Hospital of Philadelphia, Philadelphia, PA, USA; 47). 22q and You Center and Division of Genetic and Genomic Medicine, Children’s Hospital of Philadelphia, Philadelphia, PA, USA; 48). Department of Pediatrics, Perelman School of Medicine of the University of Pennsylvania, Philadelphia, PA, USA; 49). Department of Human Genetics, KU Leuven, Leuven, Belgium; 50). Center for Human Genetics, University Hospitals Leuven, Leuven, Belgium; 51). Mental Health & Neuroscience Research Institute, Maastricht University, Maastricht, The Netherlands; 52). Department of Psychiatry, The Hospital for Sick Children, Toronto, ON, Canada; 53). Department of Psychiatry, Temerty Faculty of Medicine, University of Toronto, Toronto, ON, Canada; 54). Program in Genetics and Genome Biology, SickKids Research Institute, The Hospital for Sick Children, Toronto, ON, Canada; 55). Department of Psychiatry, University of Toronto, Toronto, ON, Canada; 56). Dalglish Family 22q Clinic, Toronto General Hospital, Toronto, ON, Canada; 57). Clinical Genetics Research Program, Centre for Addiction and Mental Health, Toronto, ON, Canada; 58). Veterans Affairs Cooperative Studies Program Clinical Epidemiology Research Center (CSP-CERC), Veterans Affairs Connecticut Healthcare System, West Haven, CT, USA; 59). Department of Medicine, Yale School of Medicine, New Haven, CT, USA; 60). Department of Psychiatry, University of Miami Miller School of Medicine, Miami, FL, USA; 61). Bruce W. Carter Miami Veterans Affairs (VA) Medical Center, Miami, FL, USA; 62). Centre Hospitalier Universitaire Sainte-Justine Research Center, Montreal, QC, Canada; 63). Department of Pediatrics, University of Montreal, Montreal, QC, Canada; 64). Nuffield Department of Population Health, University of Oxford, Oxford, UK; 65). Institute for Molecular Bioscience, The University of Queensland, Brisbane, Queensland, Australia

## Abstract

Complex traits arise from the combined effects of rare and common genetic variation, development and environment, but resolving their joint contributions has been limited by statistical power. Here, we meta-analyze effects of recurrent copy number variants (CNVs), polygenic scores, sex, age and medications on height and body mass index in 1,447,001 individuals across 6 biobanks and clinical cohorts. CNVs show largely mirror dose-dependent effects of deletions and duplications on both traits, but a subset of loci exhibit asymmetric dose-responses on adult height, consistent with buffering of one allele but not the other. Polygenic background and medications combine with CNVs in ways broadly consistent with additivity. However, detailed analyses of loci at 16p11.2 and 22q11.2 reveal context-dependent effects that vary across development, physiology and sex. At 22q11.2, the net effect of a CNV reflects opposing and reinforcing contributions of multiple genes, providing a potential mechanism for buffering of dosage effects. These results indicate that genetic effects follow additive patterns in aggregate, while context-dependent deviations are widespread for specific loci.

Complex traits such as height^1–3^ and body mass index (BMI)^1,4,5^ are shaped by many genetic and environmental influences, both rare and common. Yet the principles by which these factors combine within individuals remain poorly understood. Genome-wide association studies (GWAS) have characterized polygenic architecture,^6–9.^ Rare variants, such as copy number variants (CNVs)^10–12^, loss-of-function^13–15^ and damaging missense^13^ variants, also have a significant influence. Research has begun to explore the effects of rare and common variants with each other^16–19^ and with the environment^20,21^. The major problem has been the limitation in statistical power. Common variants cover a vast search space with individually modest effects, while rare variants of large effect are found in very small numbers within individual datasets, limiting power to estimate effect sizes and interactions.

Recurrent copy number variants (CNVs) provide one solution to this problem. Generated by non-allelic homologous recombination (NAHR), recurrent deletions (DELs) and duplications (DUPs) repeatedly perturb gene dosage at the same genomic locus in both directions^22^. At some genomic regions, multiple breakpoints generate nested or overlapping CNVs, resulting in dosage perturbations of adjacent loci individually and in combination. This modular architecture enables a systematic dissection of how genes act in combination to influence complex traits. Lastly, the high per-locus mutation rates of recurrent CNVs result in a comparatively high population prevalence of these large-effect variants^23^, and their detectability with SNP-genotyping arrays allows population-scale studies, enabling greater power to detect effects.

This study investigates how recurrent copy number variants (CNVs) influence height and body mass index (BMI) and how their effects vary within the context of polygenic background, sex, development and environment. To this end, we performed a federated meta-analysis of CNV and SNP genotypes, where a harmonized analysis workflow for CNV genotyping, polygenic scoring, phenotype tabulation, scaling, and association testing was applied across five biobanks: UK Biobank (n = 341,425), MyCode (n = 110,497), Million Veteran Program (n = 587,744), Estonian Biobank (n = 63,220), All of Us (n = 344,075), and clinically-ascertained cohorts (N = 9,652), for a total sample of 1,447,001 individuals. (Fig. 1).

## Rare CNVs have large, dose-dependent effects on BMI and Height

Genome-wide analysis of CNVs^24,25^ typically aggregates signals across genes or regions, collapsing diverse rare alleles into a single test and limiting the ability to resolve the effects of gene dosage. Here we targeted a predefined set of 94 recurrent DEL and DUP alleles at 47 CNV hotspots that are readily detectable by all genotyping platforms (Supplementary Table 1), and many of which have associations with a variety of health or developmental conditions^26^. A focused approach provided precise estimation of genetic effects, reduced complexity of analysis workflows, and maximized statistical power (see Supplementary Note 2). We observed 81 CNV alleles at 47 loci in at least 10 subjects in our combined sample and were detected at comparable frequencies in all five biobanks (Supplementary Table 2) (excluding CLIN cohort contained participants which were ascertained on CNV carrier status).

Associations with sex- and age-normalized height and BMI were tested for specific CNV alleles (see Online Methods). For height, 19 CNVs had significant effects after correction for 47 tests (23%, 12 DELs and 7 DUPs, 4 loci had effects for both alleles, Supplementary Table 3), with many more nominally significant hits. For BMI, 16 CNVs had significant effects after correction for 47 tests (20%, 8 DELs and 8 DUPs, with 5 loci having effects for both alleles, Supplementary Table 3). Additionally, 6 CNVs had significant effects on both traits. Effect size estimates were consistent across biobanks and ancestry groups (Fig 2A–B). Results show that these CNVs have comparatively large effects relative to single genes that have been implicated by WES, (Fig 2C–D), consistent with a perturbation of multiple genes having a larger effect. However, there was not a significant correlation between CNV length or the number of genes with effect size, suggesting that the effect size of a CNV depends on the specific combinations of genes within it (**Extended Data Fig. 8**).

For both traits, effects of gene copy number were broadly dose-dependent, as evident from a dose-response curve showing a negative correlation of effect sizes for DELs and DUPs across all loci that were powered for both (N >10 carriers for each allele, Fig. 2E–F). Comparison of model fits showed that dose responses for height consisted of 2 (linear) components (Fig. 2E, **Fig. S1**), one with a “mirror” dose-dependent effect of DEL and DUP and another consisting of loci where effects were negative for one allele and null for the other. This asymmetry suggests that a subset of loci for height are permissive of a negative effect for one allele (DEL or DUP), but are buffered against a positive effect of the other. By contrast, model fits for BMI were consistent with a single linear dose-response curve (**Fig. SZ)**.

As expected, recurrent CNVs explain a small fraction of the overall heritability in the population. The CNV loci described here explained 0.23% and 0.18% of the variance in height and BMI respectively (see Methods)(Supplementary Table 4). This is less than the heritability explained by the genome-wide burden of rare gene variants, which is estimated at 3.7% and 1.2% for height and BMI^27^, respectively, but it is a non-trivial proportion considering that the CNVs investigated in this study occur in only 3% of our sample and collectively involve just 1.31 % (42 Mb) of the genome.

## Combinatorial effects of rare variants, polygenic scores (PGS) and psychiatric medications

Rare and common variants act in combination to influence complex traits such as intellectual disability^28^, autism spectrum disorder^17^, schizophrenia^16,28^, and heart disease^18^. In this study, accounting for the combination of CNV genotype and PGS explained substantial variation. For instance, upon stratifying the 16p11.2 BP4-BP5 locus by PGS quartile, the combined effects explain a range of 19.76 cm for height (Fig. 3A) and 14.27 kg/m^2^ for BMI (Fig. 3B, **table 5**), compared to a range of 10.87 cm and 6.09 kg/m^2^ (**Table 3)** for the CNV alone.

Previous studies have characterized the combined effect of rare and common variants. Penetrance and age-at-onset of pathogenic variants in *HNF1A, HNF1B,* and *HNF4A* for type 2 diabetes varied by PGS strata across multiple cohorts^29^. Other studies that have looked at the joint effects of rare and common variants in cancer^30–34^ and cognitive traits^16,17,19,35,36^ confirm that risk within a rare group is higher for the subset with high PGS, but statistically meaningful interactions have not been found.

Our combined data provides unprecedented power to test for non-additive effects of CNVs and PGS (Supplementary Note 2). We tested CNV×PGS interactions for the 32 recurrent CNVs in our study that exceeded 200 carriers in the combined cohort. A variety of weak interactions (P<0.05) were observed in single cohorts (8 for height and 5 for BMI), but did not replicate in other cohorts or in the meta-analysis (Fig. 3C–D). One nominal association in the meta-analysis (2q13 *NPHP1* DUP × PGS-BMI) was not significant after multiple test correction for 32 tests. We did observe better model fit for models that included interaction terms (**Table 4**); however, the magnitude of these interactions are in general too small to be reliably detected even in a sample of 1.4 million. Thus, the combined effects of CNVs and PGS on complex traits were largely consistent with an additive model.

A number of recurrent CNVs confer susceptibility to adult psychiatric conditions, including major depression, bipolar disorder, and schizophrenia^24^. In turn, many treatments for these disorders, such as antidepressants^37^, mood stabilizers, and antipsychotics^38^ can promote weight gain, potentially exacerbating the effect of a CNV on BMI. We extended this approach to investigate the combined effects of genes, PGS and psychiatric medications (See Online methods for description of how medication data was tabulated, see Supplementary Table 8 for medication definitions). While CNV effects on BMI were not mediated by medication use (**Extended Data Fig. 3)**, these three classes of medications were prescribed more frequently to CNV carriers than to the broader cohort. The prevalence of reported lifetime use of antidepressants and mood stabilizers was greatest for the CNVs that contribute to increased BMI (**Fig. 3E)**. However, we did not detect significant gene-environment interactions. The influence of medication on BMI was comparable across CNVs grouped by main effect (Sup **Fig. 3G**) and across quartiles of PGS (Sup **Fig 3H**). Nonetheless, the additive combinatorial effects of CNV, PGS and medications were substantial. Using the example of 16p11.2 BP4-BP5, when stratifying subjects by psychiatric medications, PGS and CNV genotype, their combined effects explained a further 1.227 kg/m^2^ difference in BMI. (Fig. 3F).

## Female-biased genetic effects of rare variants on BMI

Previous studies have identified rare and common variants with sex-specific effects, including SNPs that show stronger effects on body fat distribution in females than in males^39,40^. Sex-stratified analyses of rare variants have also identified female-specific effects^19,41^. For a subset of CNVs with sufficient sample size (>1000 carriers), we have adequate power to detect modest sex-dependent effects (<0.1 SD; Supplementary Note 2), but for most CNVs, sex differences can only be assessed in aggregate.

We compared sex-stratified CNV effects on height (Fig. 4A) and BMI (Fig. 4B). For height, CNV effects were highly correlated between sexes, with a slope not significantly different from 1 (slope = 0.879, p = 0.143), indicating homogeneous effects in males and females (Fig. 4A). For BMI, effects were also strongly correlated in males and females but showed a slope significantly less than 1 (slope = 0.707, p = 7.94×10^−8^), with many CNVs having stronger positive effects in females. While most individual CNV × sex differences were too small to detect (Fig. 4C–D), one (15q11.2 BP1–BP2 DEL) showed a gene × sex interaction on BMI that was significant after Bonferroni correction (Fig 4D). Additionally, when collapsed by effect size, CNVs with positive and neutral effects on BMI had significantly stronger positive effects on BMI in females (p = 4.4e-4 for positive CNVs, p = 5.0e-3 for neutral CNVs) (Supplementary Table 9). These results are consistent with a modest female-biased effect of rare variants on BMI.

## Age-dependent effects of CNVs on height are attributable to divergent causal pathways

The results described above represent the effects of recurrent CNVs that are detectable in an adult population. However, phenotypic characterization of CNVs is done almost exclusively in clinically-ascertained pediatric cohorts, which may not be representative of traits in adults. For instance, our results showing a mirror dose-dependent effect of 16p11.2 BP4-BP5 CNVs on height contrast with a previous study in a pediatric sample (ages 5–18) that showed that both the deletion and duplication were associated with short stature^42^.

Using cross-sectional data on participants from our clinically-ascertained cohorts and All of Us, we investigated whether the effects of 16p11.2 CNVs varied across the lifespan. We observed that the effects of the 16p11.2 BP4-BP5 CNVs differed dramatically by age. 16p11.2 BP4-BP5 DUP had divergent effects on height across development consisting of shorter stature as children and taller stature as adults (Fig. 5A). The growth trajectory of the reciprocal 16p11.2 BP4–BP5 DEL varied to an even greater extent. The DEL was associated with reduced height early in childhood and again in adulthood, but the trajectory showed transient tall stature between ages 8 and 13. Thus, the conflicting findings of this study and previous reports^42^ are likely explained by age-dependent effects on height (Fig. 5B). Likelihood ratio tests comparing nested generalized additive models showed that including a smooth function of height by age significantly improved model fit relative to a null model for both DUP (p=4.51e-5) and DEL (p=5.34e-11) indicating a significant age-dependent effect. Effects of 16p11.2 CNVs on BMI were also age-dependent, but the interaction was weaker (**Extended Data Fig. 4)**.

Previous research has shown that childhood obesity can influence growth in 16p11.2 deletion syndrome^43^ and in other monogenetic forms of obesity^44^. We investigated two hypotheses to explain age-dependent effects of 16p11.2 BP4–BP5 CNVs on height: first that CNVs have direct effects that vary by age, or second that age-dependencies are attributable to indirect effects on growth mediated by childhood obesity. CNV carriers were split evenly into lean and obese groups by a cutoff at the 90th percentile of BMI (based on population norms). When there were very few obese subjects (for example, the 16p11.2 BP4-BP5 DUP), a less stringent threshold was used to more evenly split the population. For 16p11.2 BP4–BP5 DUP (Fig. 5C), age-dependent effects on height did not differ by BMI group (p = 0.451). For the DEL (Fig. 5D), the steep growth trajectory was entirely restricted to subjects with obesity (BMI >90th percentile) with a height–age relationship that was significantly different than for subjects without obesity (p = 9.18×10^−4^). This differential height-age relationship between subjects with obesity and subjects without obesity was present in both female and male 16p11.2 BP4-BP5 DEL carriers in sex stratified analyses (**Extended Data Fig. 5**). No significant differences were observed when stratified by PGSs (**Extended Data Fig. 5**). The absence of age-dependent effects for PGS-BMI and PGS-Height further supports that these patterns reflect true CNV × age and CNV × BMI × age interactions, rather than artifacts of population stratification in cross-sectional data.

We then performed cross-sectional mediation analysis across developmental stages to differentiate direct CNV effects (CNV → height) from indirect effects (CNV → BMI → height). Indirect effects were observed for both DUP (Fig. 5E) and DEL (Fig. 5F) during early adolescence. Testing the reverse pathway (CNV → height → BMI) showed no indirect effects for DEL, supporting a true BMI-mediated effect. For DUP, nominal reverse effects suggested the observed mediation may reflect correlation between height and BMI rather than causation. Together, these findings suggest that the DUP has age-dependent effects on height that are mostly a direct effect, and age-dependent effects of the DEL consist with a combination of negative direct effects on height and transient positive effects mediated by childhood obesity.

**Figure 1:**
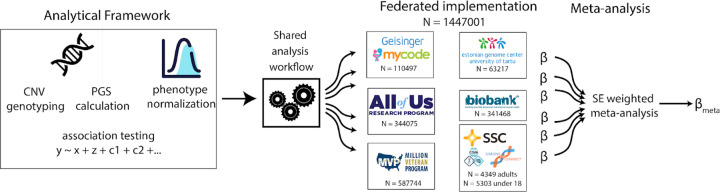
Federated biobank meta-analysis of effects of recurrent CNVs and PGS on height and BMI

Age-dependent effects of CNVs on height were then replicated in a clinically-ascertained sample of 22q11.2 A-D DUP (N = 210) and 22q11.2 A-D DEL (N=531) (**Extended Data Fig. 6)**. In contrast to its association with short stature in adults (Fig. 1), the 22q11.2 DUP was associated with tall stature in children (Fig. 5A, p = 0.00012). Likewise, associations of the DEL with short stature varied by age with a stronger effect in children (Fig. 5B, p = 0.0258). The DEL effect on Height also showed a significant interaction with BMI (Fig. 5D). Our results demonstrate that, for both loci 16p11.2 and 22q11.2, age-dependent effects were attributable to similar mechanisms. Duplications exhibit divergent direct effects on stature in children and adults, while deletions are subject to opposing effects of multiple causal pathways including (1) direct (negative) effects of the deletion and (2) transient (positive) effects mediated by childhood obesity.

## The influence of a CNV represents multiple genes with opposing or reinforcing effects

**Figure 2:**
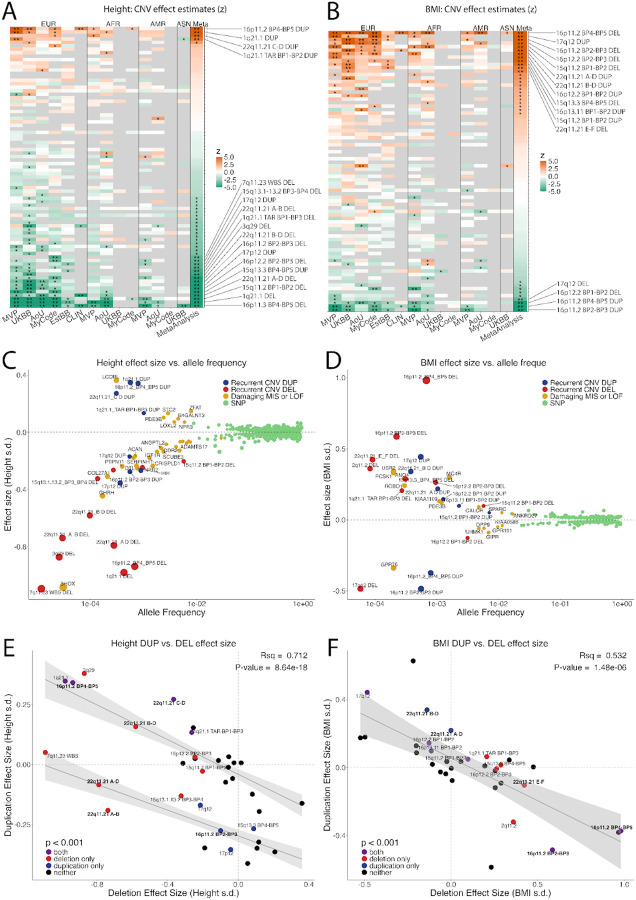
Main effects of recurrent CNVs on body mass index and height. CNV effect sizes (z) for (**A)** height and (**B)** BMI across biobanks and in meta-analysis. **significant after correction for 47 loci, *p < 0.05. Recurrent CNV associations with (**C)** height and (**D)** BMI from the present study (Supplementary Table3) in context with SNPs^1 3^, and rare pLOF or damaging missense variants^14,13^. The lead GWS SNP in each 500kb window is shown. Dose-response curves showing correlation of effects sizes of duplication vs. deletion for (**E)** height and (**F)** BMI, which includes 34 loci that had sufficient power (N>10 carriers for both DEL and DUP) to estimate both. Labeled points represent bonferroni significant associations. Summary statistics for panels A-F are in Tables S2 and S3. (E) Recurrent alleles of the 22q11.2 locus are labeled in **bold** to highlight those with mirror effects (22q11.2 C-D and B-D) and asymmetric effects (22q11.2 A-B, A-D) which are described in greater detail in Figure 6.

We further investigated the basis of asymmetric dose responses on height for the 22q11.2 A-D locus (Fig. 1E). Its modular genomic architecture enables us to dissect the combinatorial effects of three subregions, each separated by a dense cluster of segmental duplications (Fig 2A). NAHR rearrangements can occur between any combination of breakpoints A, B, C and D^45^, thereby producing DUPs or DELs of the A-B (28 genes), B-C (5 genes) and C-D (12 genes) subregions or multiple adjacent loci (A-C, B-D, A-D)^46^. By comparing gene dosage effects of each locus individually and together, we can quantify their combinatorial effects.

Previous studies in small cohorts and case reports have reported that 22q11.2 DUPs exhibit both short stature and overgrowth^47–49^. Here, we show that DUPs of each of the three subregions have distinct effects on height: A–B DUPs were associated with reduced stature, B–C DUPs had no effect, and C–D DUPs were associated with increased stature (Fig. 6B). When the neutral B–C interval was co-duplicated with either A–B (Fig. 6C) or C–D (Fig. 6D), the combined effect was neutral (similar to the largest individual effect). For DUPs spanning all three segments (A–D), the opposing effects of A–B and C–D offset each other, resulting in an attenuated net effect of the A-D DUP on height (Fig. 6E–F).

The effects of 22q11.2 DELs on height have been well characterized previously^46,50–55^. Consistent with these studies, DELs were associated with reduced stature (Fig. 6G), and we show that the combined effect of A–B and B–C DELs (A–C) was consistent with an additive model (Fig. 6G). However, the effect of deletions spanning the full region (A–D) was weaker than expected under additivity (Observed vs. Expected, P = 0.01; Fig. 6H). While the mechanism underlying this sub-additive effect is unclear, it parallels the buffering observed for duplications, suggesting that partially opposing effects among genes within the locus may also attenuate the net impact of deletions on growth.

Analysis of BMI at the same locus reveals a complementary pattern. For DUPs, subregions largely showed convergent, reinforcing effects consistent with additivity (**Extended Data Fig. 7)**. In contrast, DELs of the A–B and C–D subregions had opposing effects on BMI (**Extended Data Fig. 7C)**, and when combined in the full A–D DEL, these effects partially neutralized one another (**Extended Data Fig. 7I**). Together with the opposing effects observed for duplications on height, these results suggest a buffering of the phenotypic effects of a CNV can arise due to a combination of gene effects with opposing directionalities. These results highlight a potential mechanism by which gene dosage effects are constrained.

## Discussion

A sample of this scale—1.4 million individuals—provides the statistical power required to examine how rare and common variants, together with developmental and environmental factors, shape complex traits. By leveraging recurrent CNVs as modular perturbations of gene dosage, we identify general principles governing their effects. Gene dosage shows largely mirror, dose-dependent relationships with height and BMI, and effects of these CNVs combine additively with polygenic background and medication use. However, analyses within specific contexts reveal a more complex biology, including asymmetric dose responses consistent with buffering, as well as sex-dependent effects on BMI, age-dependent effects on growth, and modulation by physiological state such as obesity.

Recurrent CNVs represent among the largest-effect variants identified in genome-wide studies. The median un-signed effect size for recurrent CNVs was 0.341 SD (2.56 cm for males) compared to 0.177 SD (1.328 cm) for rare gene variants and 0.023 SD (0.172 cm) for common SNPs^57^. Our results are consistent with large-scale copy number changes of multiple genes having large combined effects. However, no clear relationship was found between the number of genes within a locus and its effect size (**Extended Data Fig. 8**); thus, the net effect of a CNV is dependent on the particular combination of genes within it.

While most loci follow a linear “mirror” dose-response with DEL and DUP typically having effects in opposite directions, a subset of loci for height have effects that are asymmetric and biased in the negative direction (short stature), suggesting that the effects of some gene clusters are tightly buffered against positive effects on growth. This result is consistent with another preprint by Milind et al.^58^, which found evidence that non-monotonic (U-shaped) dose-response curves are common for height, such that both DELs and DUPs typically shift the trait in the negative direction.

Several mechanisms could underlie asymmetric gene-dosage effects, including negative feedback regulation, dosage compensation, and compensatory interactions within gene regulatory networks^59^. A model proposed by Milind et al.^58^ hypothesized that buffering is intrinsic to the trait, due to the manner in which the trait responds to diverse forms of upstream dysregulation^58^. This theory likely could explain non-monotonic effects of CNVs on traits such as cognitive performance where reciprocal DELs and DUPs at the same locus are both associated with developmental delay^60^. However, results in this study support a different mechanism for height (see Supplementary Note 3 for discussion of results in context of Milind et al.). In our data, only a subset (~30%) of loci have asymmetric effects, and only a fraction of these can be described as non-monotonic (e.g. 22q11.2 A–B and A–D, Fig. 6). We show that buffering can arise from the aggregate behavior of multiple genes within a locus. When opposing effects of the 22q11.2 A-B and C-D DUPs are combined within the full A-D DUP, there is an additive cancelation of opposing effects (Fig. 6F). When reinforcing negative effects of the A-B and C-D DELs are combined with the full A-D DEL, the net effect is sub-additive (Fig. 6I), suggesting that gene dosage effects can also be attenuated in the negative direction. Together, our findings suggest that buffering can operate in both directions through combinations of reinforcing and opposing gene effects. Because many CNVs in the population span multiple adjacent genes, it is likely that some non-monotonic effects of specific loci described by Milind et al. involve the combinatorial effects of multiple genes.

CNV effects on height were strongly age-dependent, with opposing directional effects in childhood and adulthood. Growth trajectories inferred from cross-sectional data revealed consistent patterns across reciprocal CNVs at both 16p11.2 and 22q11.2. DUPs at both loci showed direct age-dependent effects of genes. At 22q11.2, opposing effects of the A-B and C-D subregions suggest a possible mechanism for age-dependent effects if the A-D DUP: if the effects of genes within the A-B and C-D intervals act at different stages of development, this could produce a positive effect of the A-D DUP in childhood and a negative effect in adulthood. Age-dependent effects of DELs at both loci appear to reflect a combination of causal mechanisms, including negative direct effects on growth and transient positive effects mediated by childhood obesity^61,44^. This pattern—early overgrowth followed by a plateau—resembles precocious puberty^62^, a phenotype also associated with 16p11.2 deletion syndrome^63^. Together, our findings support a model in which age-dependent CNV effects arise from the interplay of direct gene dosage effects on growth with indirect effects of the CNV mediated by metabolic and hormonal pathways.

We observe modest but consistent sex differences in CNV effects on BMI, with stronger positive effects in females, consistent with findings reported in a subset of this sample^19^. By contrast, a consensus from GWAS has found that SNP effects on height and BMI are generally homogeneous between the sexes^39,64^. Population-level studies of whole genomes or exomes on a similar scale are needed to determine if sex differences are a general property of rare variants of large effect.

While the observed additive effects of polygenic background and medication were not surprising, they were nevertheless informative about the predictability and biological basis of these complex traits. When CNV carriers were stratified by polygenic scores (PGS) the combined effect sizes of the 16p11.2 BP4-BP5 CNV and PGS spanned a range of 19.76 cm for height and 14.27 kg/m^2^ for BMI. These results illustrate how polygenic background shifts the baseline on which large-effect variants act, substantially expanding the range of phenotypic outcomes among carriers.

**Figure 3.**
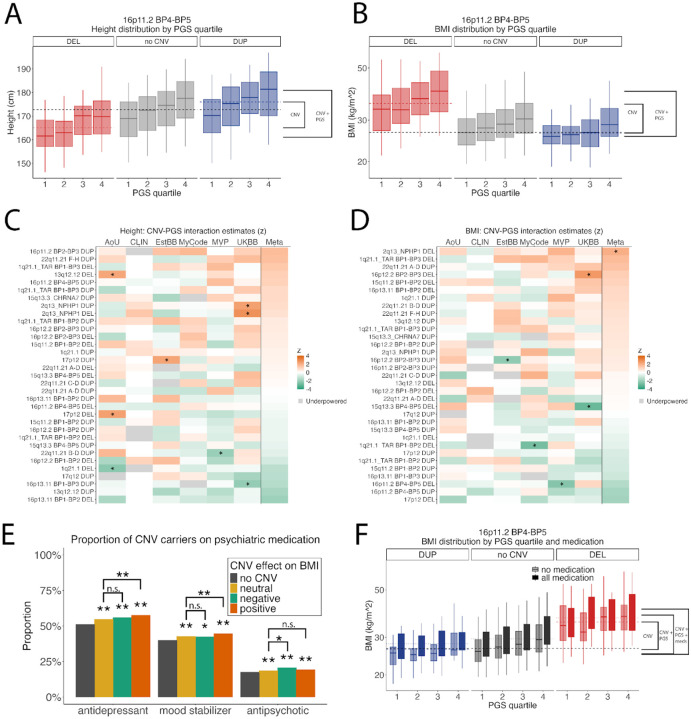
The combined effects of CNV and PGS and medication on complex traits. We also observe wide. variation in traits when we stratified the 16p11.2 locus by CNV genotype and PGS quartile, (A) spanning a range 19.76 cm for height and (B) 14.27 kg/m^2^ for BMI. C-D) Heatmap of z-transformed interaction term estimates in each biobank and the meta-analysis. * = p < 0.05. (E) Proportion of individuals who reported lifetime use of three broad classes of medication, in CNV carriers and non-CNV carriers. ** = p < 0.001 for Chi2. (F) BMI of 16p11.2 BP4-BP5 CNV carriers stratified by PGS quartile and medication. The data used to generate this figure can be found in Supplementary Tables 5 and 6.

**Figure 4:**
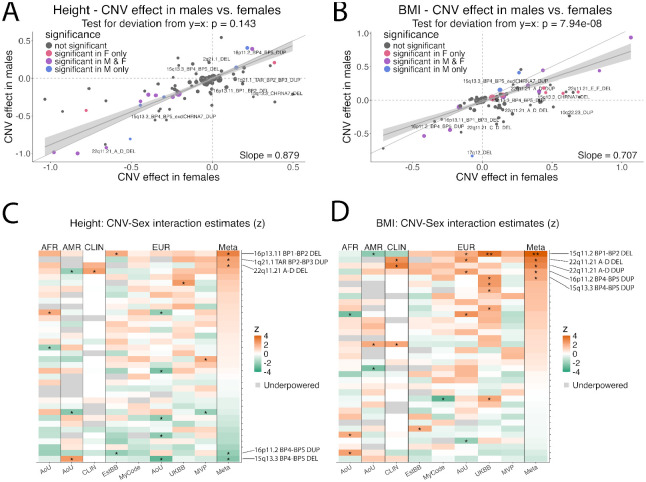
Sex stratified analysis of CNV effects on height and BMI. Correlation of CNV effect sizes in males and females for (**A**) Height and (**B)** BMI. Deviation from y=x was tested via F-test of a joint linear hypothesis. Heatmap of z-transformed interaction term estimates from linear regression in each biobank and the meta-analysis for **C)** Height and (**D)** BMI. Cohorts with significant sex imbalance (such as MVP, 80% male) were excluded. CNVs with under 200 carriers were excluded from interaction analysis (see Supplementary Note 2 on Statistical Power). Summary statistics for panels A-D are in Supplementary Table 9

**Figure 5.**
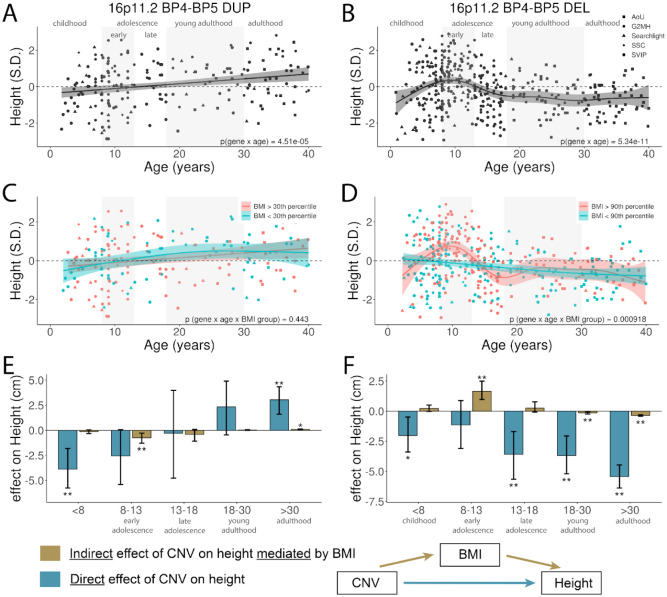
Effects of 16p11.2 BP4-BP5 CNVs differ by age. Age and sex normalized height values vs, age for (A) 16p11.2 BP4-BP5 DUP and (B) 16p11.2 BP4-BP5 DEL. C-D). Cross-sectional height trajectories further stratified by BMI at the 30th (C) and 90th (D) percentiles for DUP and DEL, respectively. E-F). Age-stratified cross-sectional mediation analysis characterizing the distinct causal pathways underlying CNV effects on height, for DUP (E) and DEL (F). Z scores represent height normalized by age and sex relative to WHO norms, and deviation from zero represents the difference in height relative to the normative population. The upper end of each age range is exclusive (i.e., 8–13 represents those who are at least 8 years old but under 13 years old).

**Figure 6:**
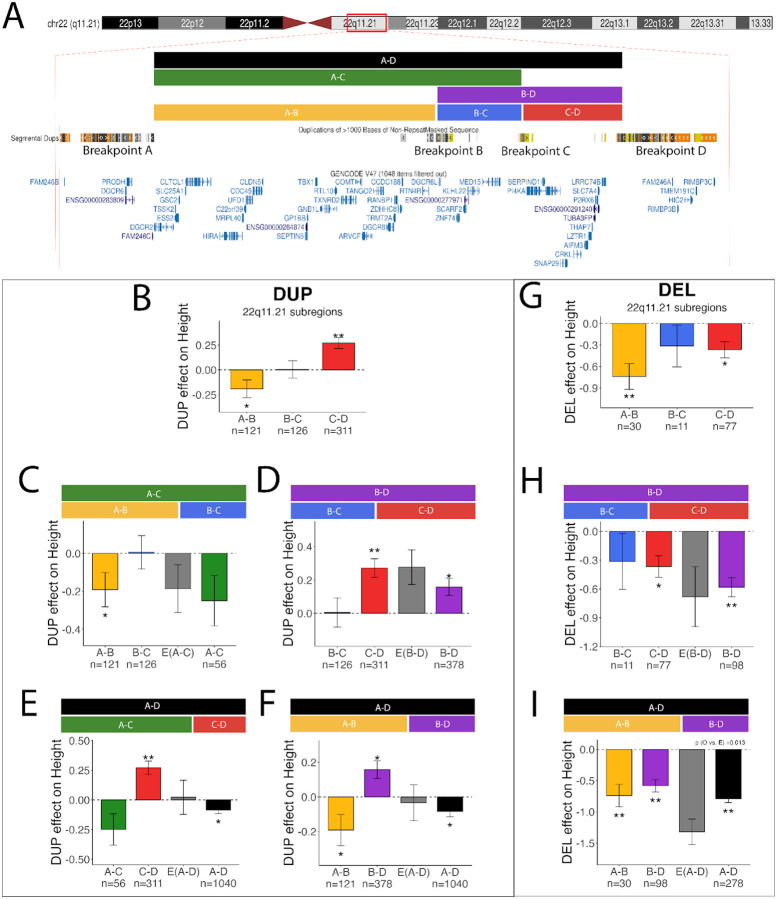
Dissection of the 22q11.2 A-D locus and its effects on height. A). Schematic of the genes encompassed by the most commonly observed CNV breakpoints in our study, as defined by Gencode V49 accessed through the UCSC Genome Browser^56^ B-C). Breakdown of the effects of DUPs (B-F) and DELs (G-I) of 22q11.2 subregions. E(A-C) denotes the expected value of the effect size of A-C based on the sum of the effects of subregions A-B and B-C. Summary statistics for panels B-I are in Table S3.

Medications further shape these effects. Consistent with the associations of recurrent CNVs with psychiatric conditions^24^, CNV carriers were more likely to be prescribed psychiatric medications, particularly those who carried CNVs that predispose to obesity, which is consistent with genetic correlations of BMI and mental health traits^65^. Given that weight gain is a side effect of many antidepressants^37^, mood stabilizers^38^ and antipsychotics^66^, our results provide an example of a pharmacogenomic effect, in which genetic susceptibility to a psychiatric condition itself influences metabolic outcomes of treatment. As we show here, the combined influences of 16p11.2 CNV genotype, PGS and psychiatric medication on BMI are quite large. The stratified group-means span a range of 15.5 kg/m^2^ (Fig. 3F), which corresponds to a range of >45 Kg for the average individual.

While this study was designed to define basic principles governing the combinatorial effects of genes in humans, it also has significant clinical implications. Many CNVs studied here are routinely reported in clinical genetic testing (Supplementary Table 1) and are associated with developmental, mental health and metabolic conditions^19^. A broad understanding of how CNVs, polygenic background, sex, development and medications jointly shape phenotypic variation can improve the prediction of clinical outcomes and inform patient management. These insights also highlight potential avenues for therapeutic intervention of genetic conditions by identifying modifiable pathways^67^ through which gene dosage can influence development.

## Methods

### Genotyping and CNV calling

In previous large-scale studies of CNVs, we have shown that CNV detection varies significantly due to genotyping platform, but confounding due to platform-specific CNV detection is well-controlled when we apply a meta-analytic approach^24^ : (1) all data processing and statistical association tests are first performed within platform, then (2) multiple platforms are combined by meta-analysis of summary statistics. Furthermore, large recurrent CNVs are reliably detected by virtually all commonly-used SNP genotyping platforms using relevant CNV calling software^24^, and we confirmed that recurrent CNVs were detected in this study with comparable frequencies across all cohorts (Supplementary Table 2). Thus, a federated meta-analysis of biobanks can utilize previously existing CNV call sets as input files to standardized shared analysis workflow that is developed centrally and then implemented at all sites. A key aspect of the CNV calling that must be standardized across sites is the assignment of a CNV genotype from raw CNV calls. Methods for standardized CNV genotype assignment is described here and methods for SNP genotyping and raw CNV calling in each Biobank is described below.

#### CNV genotype assignment from existing CNV calls

Each biobank used the same pipeline to generate recurrent CNV genotypes (see Code Availability). To generate recurrent CNV genotypes, we ran bedtools^68^ intersect, with the CNV calls generated by the various CNV calling methods and the recurrent CNV definitions^24^ (Supplementary Table 1) as input. For CNVs with a single set of breakpoints, we require that the observed CNV overlaps with at least 50% of the target CNV region. This corresponds to the -f.5 option in bedtools intersect. For CNV with multiple sets of breakpoints (such as the 22q11.2 region), genotypes were defined as the union of individual sets of breakpoints. For example, for an individual to have a DEL in 22q11.2 A-D region, they would have to have a CNV that spans at least 50% of the A-B, B-C, and C-D regions, and no CNV spanning the D-E, E-F, F-G, or G-H regions.

#### UKBB

Genetic data for the UK Biobank was genotyped on the Affymetrix UK Biobank Axiom Array. CNV calling on Affy Axiom arrays use PennCNV^69^ and QuantiSNP^70^. The consensus of CNV calls from multiple callers was created by merging CNVs at the sample level and retaining CNVs that were called by at least 2 methods. Sample-level and CNV-level QC was performed as previously described^24^.

#### All of Us

A total of 447,278 microarray-derived VCF files were obtained from the All of Us Research Program (https://doi.org/10.1038/s41586-023-06957-x). CNVs were identified using two algorithms: PennCNV^69^ and QuantiSNP^70^. Three samples were excluded due to missing sex information, which is required by both algorithms. Each CNV call set was pre-filtered independently to remove low-confidence variants prior to intersecting the results from both methods. Specifically, CNVs with confidence scores below 15 in either PennCNV or QuantiSNP were excluded. In addition, only CNVs larger than 1 kilobase were retained.

#### EstBB

SNP genotyping data from 205,841 Estonian Biobank (EstBB) participants were generated using the Infinium Global Screening Array (GSA; Illumina Inc.). Samples were excluded if genotype call rate was <95% or if sex inferred from X-chromosome heterozygosity was inconsistent with recorded phenotypic sex. Prior to CNV calling, genotype clusters were manually realigned to improve signal quality, and log R ratio and B allele frequency values were exported from GenomeStudio v2.0.5 (Illumina). Autosomal CNVs were called with cnvPartition v3.2.0 (Illumina Inc.), followed by sample- and CNV-level quality control. Samples with extreme CNV burden (>200 CNV calls or cumulative CNV length >10 Mb) were removed.

#### MyCode

CNVs were called from whole-exome sequencing data using CLAMMS (Copy number estimation using Lattice-Aligned Mixture Models), as described in Maxwell et al.^71^ and used in subsequent MyCode CNV analyses. SNP genotypes were available for a subset of participants generated on the Illumina HumanOmniExpressExome-8 v1.2 array^72^. A harmonized set of SNP genotypes on both platform was created using the subset of overlapping variant sites from DiscovEHR WES and the Omni chip platform.

#### MVP

SNP genotyping data for MVP participants was generated using the MVP 1.0 custom Axiom array^73^. Samples were excluded for genotype call missingness >2.5%, excess heterozygosity, potential duplication, or discordance between genetic sex and self-identified gender. Variants were excluded if missingness exceeded 5% or if minor allele frequency differed by more than 10% from 1000 Genomes Project Phase 3 reference data. All analyses were performed using the GRCh38/hg38 human genome reference build. Copy number variants (CNVs) were identified using PennCNV (v1.0.5) with the PennCNV-Affy protocol^74,75^. Raw probe intensity data were converted to log R ratio (LRR) and B-allele frequency (BAF) values using Affymetrix Power Tools, and CNVs were inferred with a hidden Markov model implemented in PennCNV.

#### G2MH

Per-sample data quality was assessed by computing the median absolute deviation (MAD) of GC-corrected coverage (WGS) or Log R Ratio (GSA) values across mappable regions (after excluding assembly gaps, segmental duplications, and simple repeats). Samples with elevated MAD were excluded. For WGS samples, CNVs were called using the DRAGEN CNV caller (v3.8.4, Illumina) and all were manually verified in WGS coverage data. For samples that were genotyped with GSA array samples, CNVs were called with iPattern and PennCNV as described previously^24^.

#### SSC

Genetic data for the SSC was genotyped on the Illumina 1Mv1, 1Mv3 and HumanOmni2.5–4v1_B microarrays. IDAT files are used to store BeadArray data and Illumina Genome Studio was used to generate genotype call (GTC) files and final report files. CNVs were called with iPattern and PennCNV, and sample-level and CNV-level QC was performed as described previously^24^.

#### Simons Searchlight

Simons Searchlight (formerly known as Simons VIP^60^) participants were clinically ascertained on CNV carrier status. Thus, we used the recurrent CNV genotypes provided by SFARI in this study.

### SNP Imputation and QC

SNP Imputation and QC followed slightly different procedures across biobanks, due to each biobank’s unique population structure and genetic data availability. Since individual level data was not combined across biobanks, and ancestry PCs and PGSs were scaled within each biobank, batch effects were not a concern.

For UK Biobank, SNPs were imputed from microarray data using the Axiom array and 1000 Genomes Phase 3 Reference panel. For MyCode, SNPs were imputed from Illumina WES data using the 1000 Genomes Phase 3 Reference panel. For All of Us, SNPs were imputed from Illumina Infinium H3Array data using the Haplotype Reference Consortium reference panel. For EstBB, Imputation was performed with Beagle v5.4 software and default settings. A population-specific reference panel consisting of 2,695 high-coverage (~30x) WGS samples was utilised for imputation^76^, and standard Beagle hg38 recombination maps were used. MVP was genotyped on MVP 1.0 custom Axiom array, and imputed using the 1000 Genomes Phase 3 Reference panel. For the Clinically Ascertained cohorts, subjects who were only genotyped with SNP microarrays (Infinium GSA-24 for G2MH, HumanOmni2.5–4v1B and Illum1M for SSC, and Affy CytoScan HD and Agilent aCGH for Searchlight) were imputed via the Ricopili^77^ pipeline, with TopMed as the reference panel. SNPs from subjects with WGS data available were then subsetted to match available imputed SNPs. Subjects with no available SNP genotype data (CNV genotypes, demographics, and phenotypes only) were only included in trajectory analyses.

### Ancestry PCA

Ancestry principal component analysis (PCA) was run separately for each biobank, within broad genetically partitioned ancestry groups (i.e, EUR, AMR, AFR, EAS). We used the flashpca^78^ tool to generate the top 10 ancestry principal components based on a set of minimally correlated SNPs, with exact numbers varying across cohorts. The top 10 PCs explained 1.8% and.25% of variance in height and BMI on average (respectively), though there was mild heterogeneity across cohorts and ancestry groups, with variance explained ranging from.18% to 13% for height and 0.106% to 1.87% for BMI. Across both traits, the AMR ancestry designation had the highest variance explained by PCs. This was true for the AMR ancestry groups in both All of Us and MVP. This is consistent with the fact that this population has a high admixture of haplotypes from broad global ancestry groups. (Supplementary Table 4).

### Polygenic risk score calculation

We used PGScs^79^ with 25K MCMC iterations, 10K burn-in and phi=0.01 to generate SNP weights based on GWAS: BMI with^4^ and without^1^ UKBB, and height with^2^ and without^3^ UKBB. For downstream analysis, we used the PGSs excluding UKBB for UKBB, and the PGSs including UKBB for all other cohorts. PGSs calculated using PLINK 1.9’s –score option. For downstream analysis, PGSs were scaled to a standard normal distribution for each cohort.

### Scaling of the response variable

For our adult subjects, we scaled our BMI and Height response variables in a manner consistent with^5^. We scaled height and BMI in each cohort separately. To scale a response variable y, we fit a model lm(y ~ sex + age + agê2), and extracted the residuals. Next, we split males and females, then applied a BoxCox transformation to achieve a normal distribution in the response variable. Finally, we recombined males and females. The BoxCox transformation allowed us to achieve normally distributed residuals when fitting out models without moving outliers closer to the mean. This ensures that CNVs with large effect sizes don’t have deflated effect size estimates. Splitting males and females when applying the BoxCox transformation and standard normal scaling ensures that the variance in the response variable is consistent across males and females. A normally distributed response variable is especially important when characterizing interactions, as a non-normal response can result in a misspecified model and lead to type I errors (see Supplementary Note 1: Model Misspecification)

For pediatric subjects, we calculated age and sex norms using the childsds R package^80^, using WHO norms for height and CDC norms for BMI.

### CNV main effect calculation

To calculate the effect size of each recurrent CNV on height and body mass index, we fit a linear regression model containing CNV genotype coded as a factor (DEL, DUP, and no CNV as the reference) and the top 10 ancestry principal components. For example, to test the effect of CNV A on BMI in a given biobank, we would fit a model:


lm(BMI~ factor(CNV A genotype, ref=No CNV) + PC1 + PC2 …. PC10, data = biobank)


We then extracted the estimated regression coefficient, standard error, and p-value for each CNV. The regression coefficient represents the mean difference in sex and age corrected BMI or height, in standard deviations. Thus, if a CNV has an estimated main effect of 1.0 on BMI, carriers of that CNV have, on average, a BMI 1.0 standard deviations higher than those of the same age and sex. Finally, we performed a fixed effects meta-analysis using the R package metafor^81^ to obtain an estimate of CNV effects across studies. We opted to use a fixed effects meta-analysis (as opposed to a random effects meta-analysis) because of the way in which the phenotypic measurements were scaled. Though there may be differences in demographics between the cohorts (for example, UKBB is older, MVP is male dominated), our scaling ensures that in each cohort, we are estimating the effect of any given variant *relative* to the rest of the cohort, which should be the same quantity across biobanks.

To calculate the variance explained by all recurrent CNVs, we fit a model containing all recurrent CNV loci coded as factors (+ covariates), and a covariate only model. We then meta-analysed R^2^ values with a 3-step process. We first applied Fisher’s transformation to the square root of R^2^ estimates. This stabilizes the variance and makes the sampling distribution approximately normal. We then applied the same fixed effects meta-analysis as above. Finally, the pooled estimate was transformed back into an R2 by reverting Fisher’s transformation and squaring the result. To obtain the variance explained by CNVs across studies, we compared the pooled R^2^ from the CNV + covariates model to the covariates only model, similar to^24^.

### Psychiatric Medication Tabulation

The psychiatric medications, their RXNorm IDs, and the broad medication categories they belong to are summarized in Supplementary Table 8. A list of medications prescribed to each individual (lifetime use when available, self-reported use when not) was extracted within each dataset, limited to the medications listed in Supplementary Table 8. Individuals were placed into the “antidepressant” category if any of the medications labeled anti-depressant were included in their list. The same procedure was followed for antipsychotics and mood-stabilizers. Finally, the “all medication” category was generated taking the union of the three sub-categories (antidepressant, antipsychotic, and mood stabilizers).

### Cross-Sectional Mediation Analysis

Mediation analysis was conducted with the mediation^82^ package in R, using raw phenotype values. The mediator model was:

BMI~sex+age+CNV

The outcome model was

HEIGHT~BMI+sex+age+CNV

The relative contributions of direct and putative indirect effects on height were estimated via bootstrap with 1000 repetitions.

## Supplementary Material

Supplement 1

1

## Data Availability

Code for shared analysis pipeline and meta-analysis can be accessed on GitHub: https://github.com/mollysacks/Sacks2026
