# Supplementary material for: Combinatorial effects of gene dosage, polygenic background and environment on complex traits
Source: medRxiv. 2026 May 1:2026.04.30.26352063. Preprint. [Version 1] doi: 10.64898/2026.04.30.26352063 (PMC13142611; doi:10.64898/2026.04.30.26352063)

medRxiv preprint doi: https://doi.org/10.64898/2026.04.30.26352063; this version posted May 1, 2026. The copyright holder for this preprint (which was not certified by peer review) is the author/funder, who has granted medRxiv a license to display the preprint in perpetuity.
It is made available under a CC-BY 4.0 International license .

Supplementary Information

Extended Data

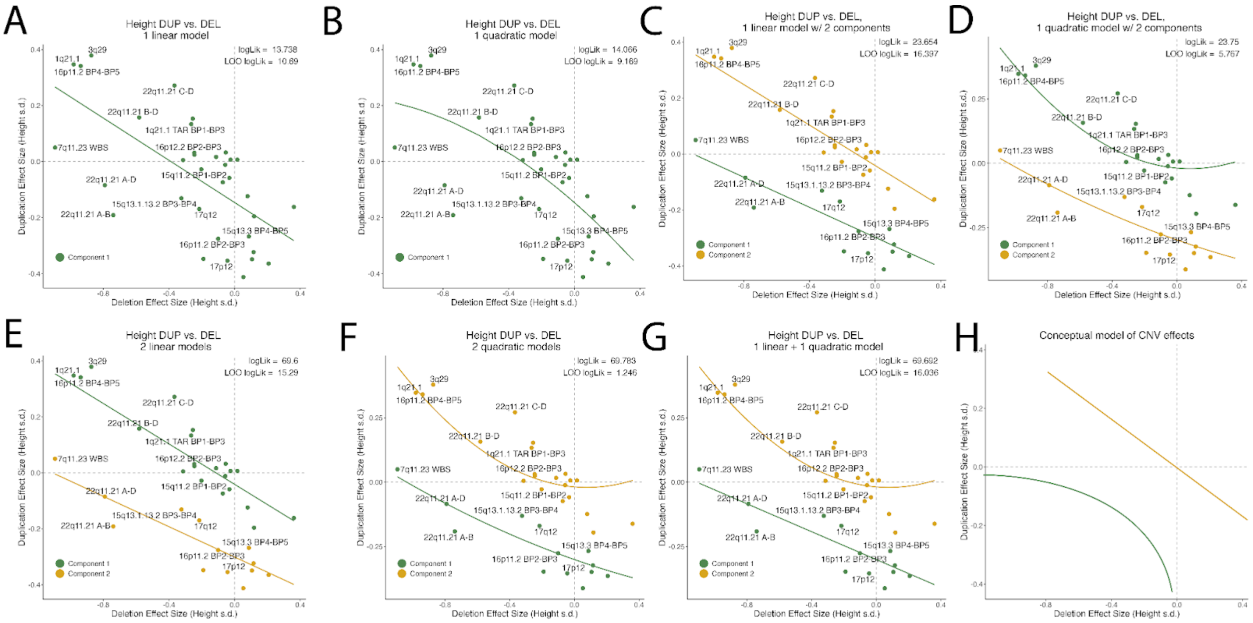

Extended Data Figure 1: Effect sizes for reciprocal deletion and duplication on Height are consistent with two dose response curves. A model consisting of a **(A)** A linear model **(B)** a quadratic model **(C)** two linear models with shared parameters **(D)** two quadratic models with shared parameters **(E)** two linear models with distinct parameters **(F)** two quadratic models with distinct parameters **(G)** a linear and a quadratic model. LOO log-likelihood was calculated for each model fit to determine out of sample prediction accuracy for each model. Panels C, E, and G had the highest LOO log-Lik, suggesting two underlying dose-response relationships **(H)** We proposed a model in which the data consist of 1 linear component and 1 non-linear component.

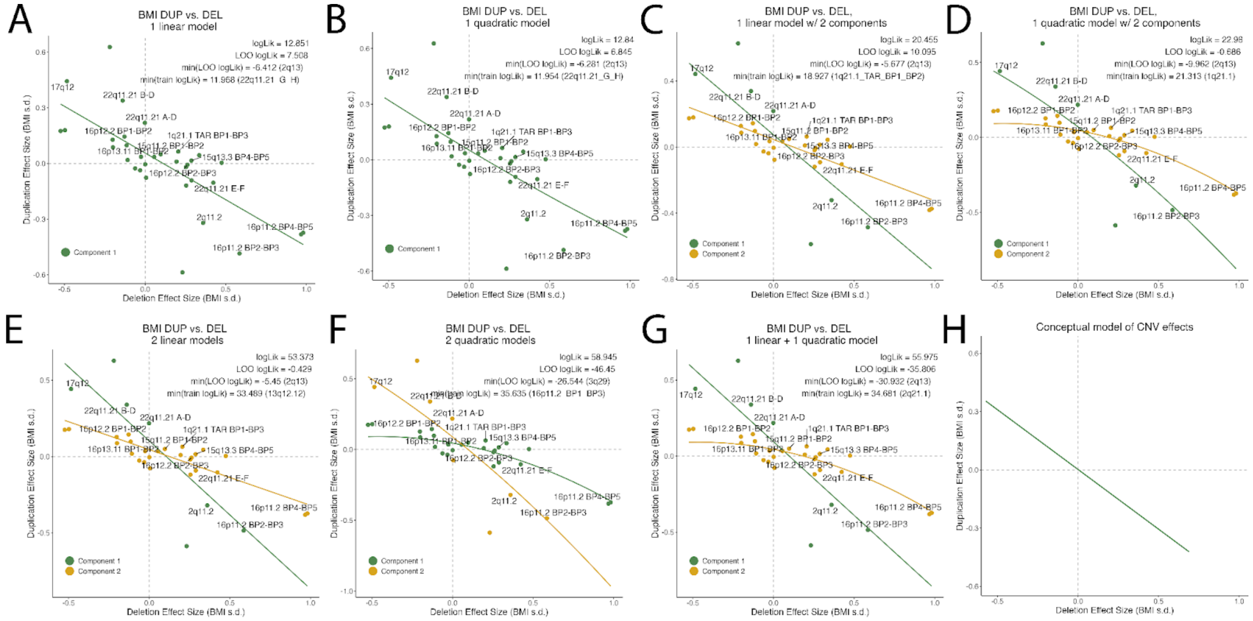

medRxiv preprint doi: https://doi.org/10.64898/2026.04.30.26352063; this version posted May 1, 2026. The copyright holder for this preprint (which was not certified by peer review) is the author/funder, who has granted medRxiv a license to display the preprint in perpetuity.
It is made available under a CC-BY 4.0 International license .

Extended Data Figure 2 Effect sizes for reciprocal deletion and duplication on BMI are consistent with a single distinct dose response curve. A model consisting of a **(A)** A linear model **(B)** a quadratic model **(C)** two linear models with shared parameters **(D)** two quadratic models with shared parameters. BIC is comparable across A-D, suggesting that there is only one underlying model. **(E)** two linear models with distinct parameters **(F)** two quadratic models with distinct parameters **(G)** a linear and a quadratic model. **(H)** We proposed a model in which the data consist of 1 linear component.

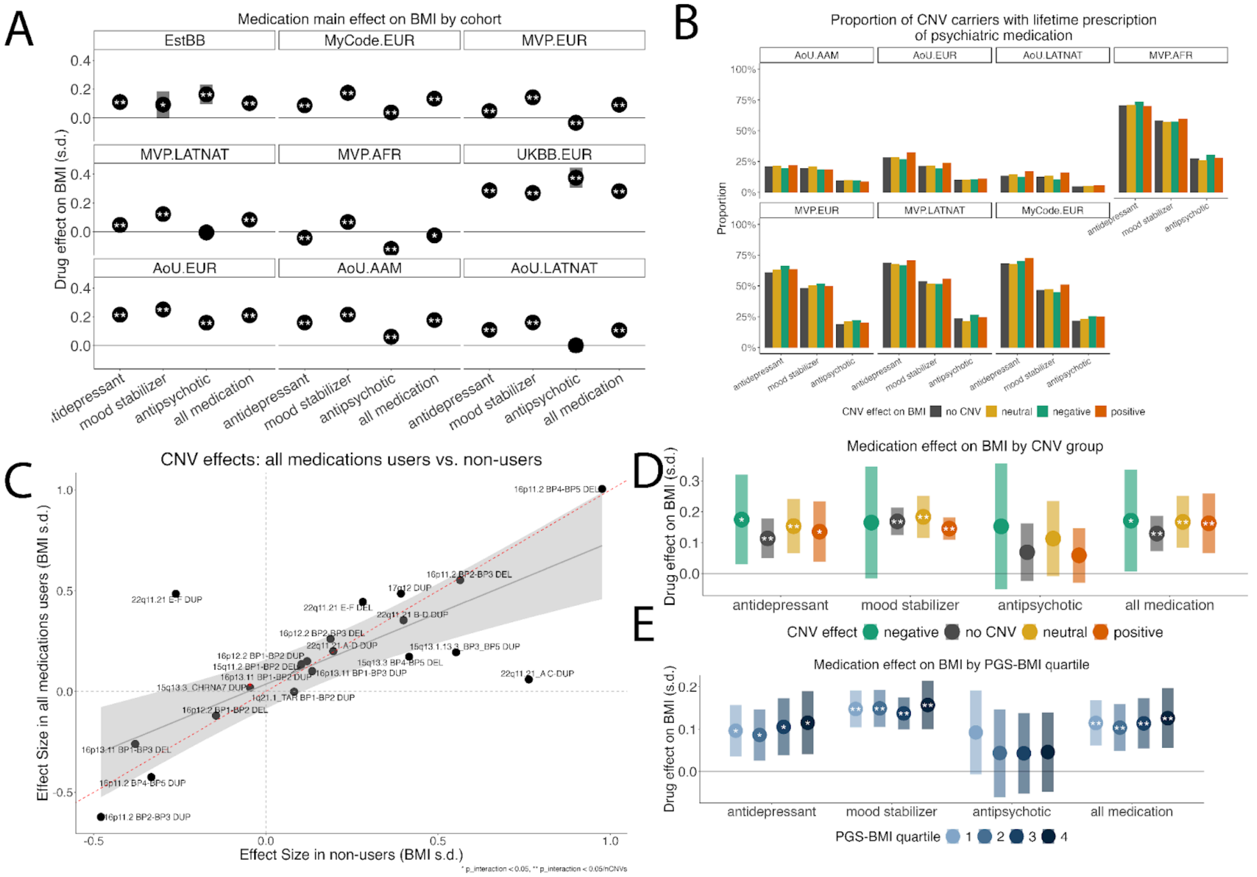

Extended Data Figure 3: Additive effects of CNVs and psychiatric medication on body mass index. A). The effect of lifetime history of use of three broad classes of psychiatric medication (and their union) on BMI in all five biobanks. B). Proportion of individuals who reported lifetime use of three broad classes of medication, in CNV carriers and non-CNV carriers, in each biobank. C). Main effect of recurrent CNVs with and without controlling for medication use. D). The effect of three broad classes of psychiatric medication (and their union) on BMI by CNV background. White asterisks represent the effect of medication in that group (* = p < 0.05, ** = p < 0.001). Black asterisks represent an interaction between CNV genotype and medication on BMI. E). The effect of three broad classes of psychiatric medication (and their union) on BMI by PRS quartile. Asterisks are the same as in D. Summary statistics for panels A-E are in Supplementary Table 6

medRxiv preprint doi: https://doi.org/10.64898/2026.04.30.26352063; this version posted May 1, 2026. The copyright holder for this preprint (which was not certified by peer review) is the author/funder, who has granted medRxiv a license to display the preprint in perpetuity.
It is made available under a CC-BY 4.0 International license .

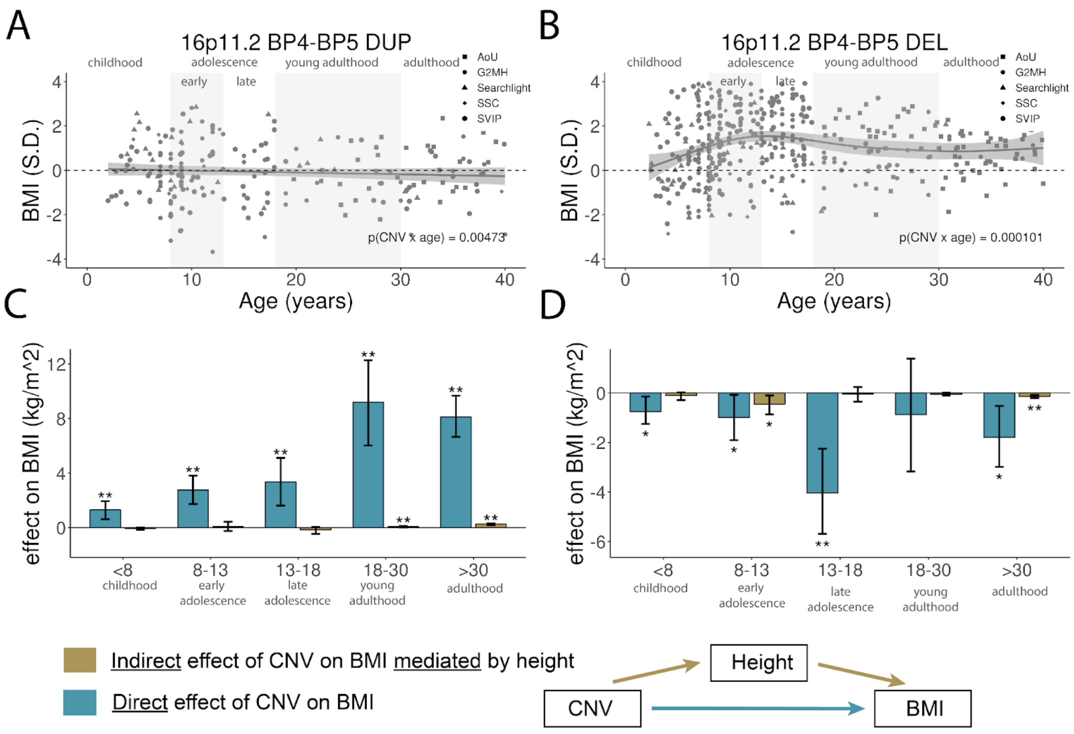

Extended Data Fig. 4: Effects of 16p11.2 BP4-BP5 CNVs on BMI differ by age. Age and sex normalized BMI values vs. age for (A) 16p11.2 BP4-BP5 DUP and (B) 16p11.2 BP4-BP5 DEL C-D). Age-stratified cross-sectional mediation analysis characterizing the distinct causal pathways underlying CNV effects on BMI, for DUP (C) and DEL

medRxiv preprint doi: https://doi.org/10.64898/2026.04.30.26352063; this version posted May 1, 2026. The copyright holder for this preprint (which was not certified by peer review) is the author/funder, who has granted medRxiv a license to display the preprint in perpetuity.
It is made available under a CC-BY 4.0 International license .

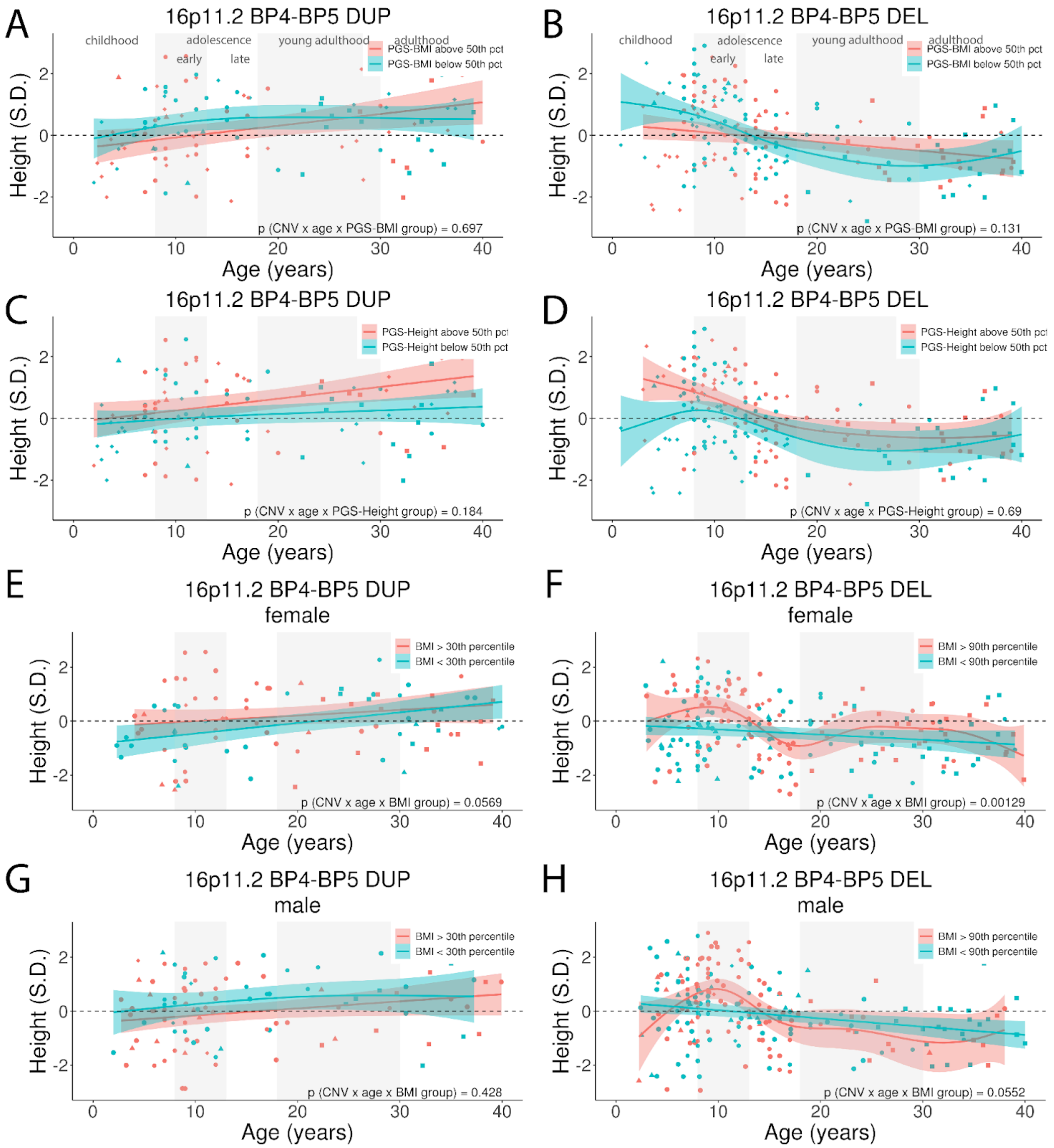

Extended Data Fig. 5. Age-dependent effects of 16p11.2 CNVs are not mediated by polygenic scores or sex. Trajectories stratified by (A-B) PGS-BMI (C-D) PGS-Height. (E-F) BMI (females only), and (G-H) BMI (males only). 16p11.2 BP4-BP5 DUP (A, C, E) and DEL (B, D, F).

medRxiv preprint doi: https://doi.org/10.64898/2026.04.30.26352063; this version posted May 1, 2026. The copyright holder for this preprint (which was not certified by peer review) is the author/funder, who has granted medRxiv a license to display the preprint in perpetuity.
It is made available under a CC-BY 4.0 International license .

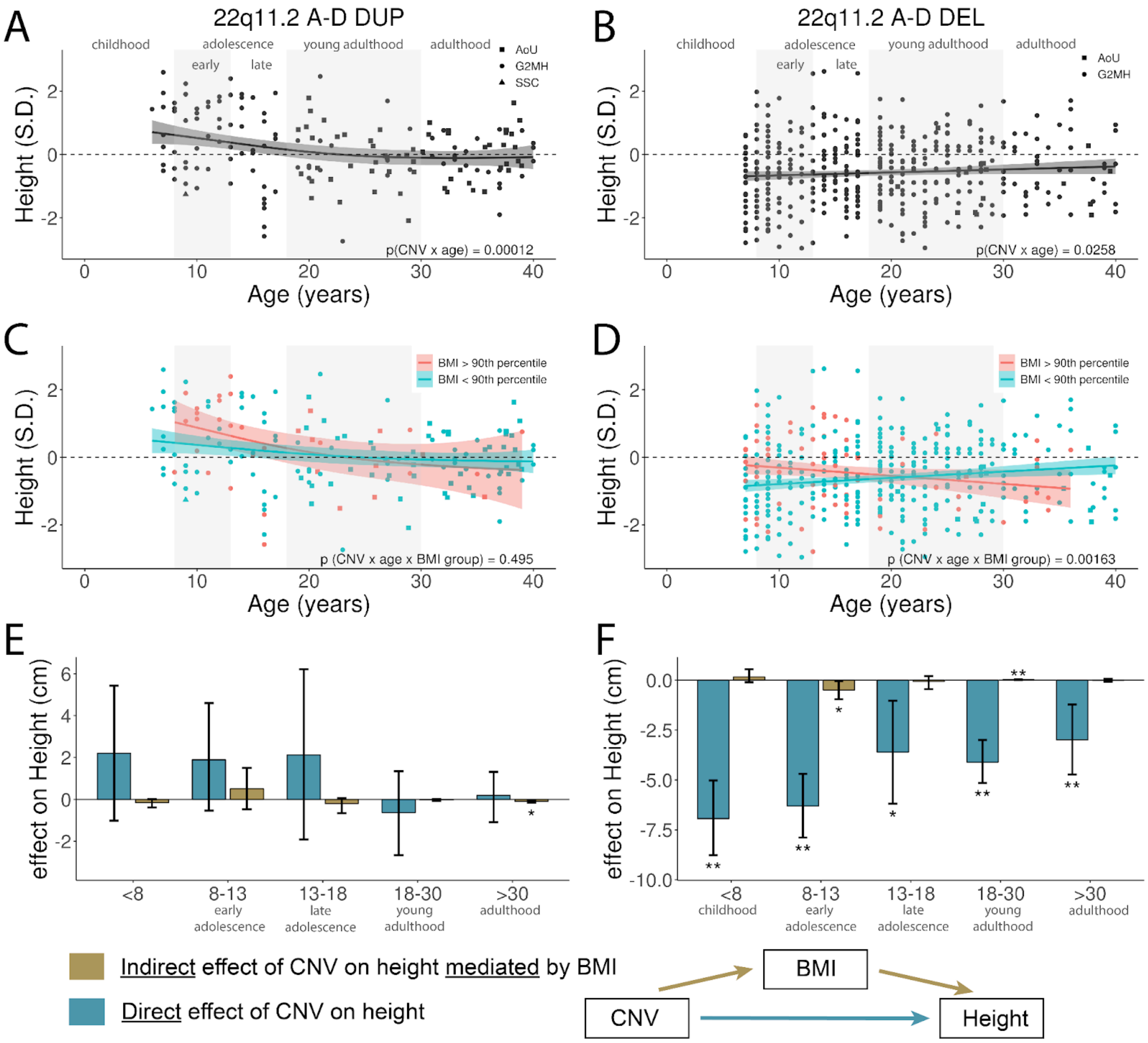

Extended Data Fig. 6: Effects of 22q11.21 A-D CNVs differ by age*.* Age and sex normalized height values vs, age for (A) 22q11.21 A-D DUP and (B) 22q11.21 A-D DEL. C-D). Cross sectional height trajectories further stratified by BMI at the 90th percentiles for DUP (C) and DEL (D). E-F). Age-stratified cross-sectional mediation analysis characterizing the distinct causal pathways underlying CNV effects on height, for DUP (E) and DEL (F).

medRxiv preprint doi: https://doi.org/10.64898/2026.04.30.26352063; this version posted May 1, 2026. The copyright holder for this preprint (which was not certified by peer review) is the author/funder, who has granted medRxiv a license to display the preprint in perpetuity.
It is made available under a CC-BY 4.0 International license .

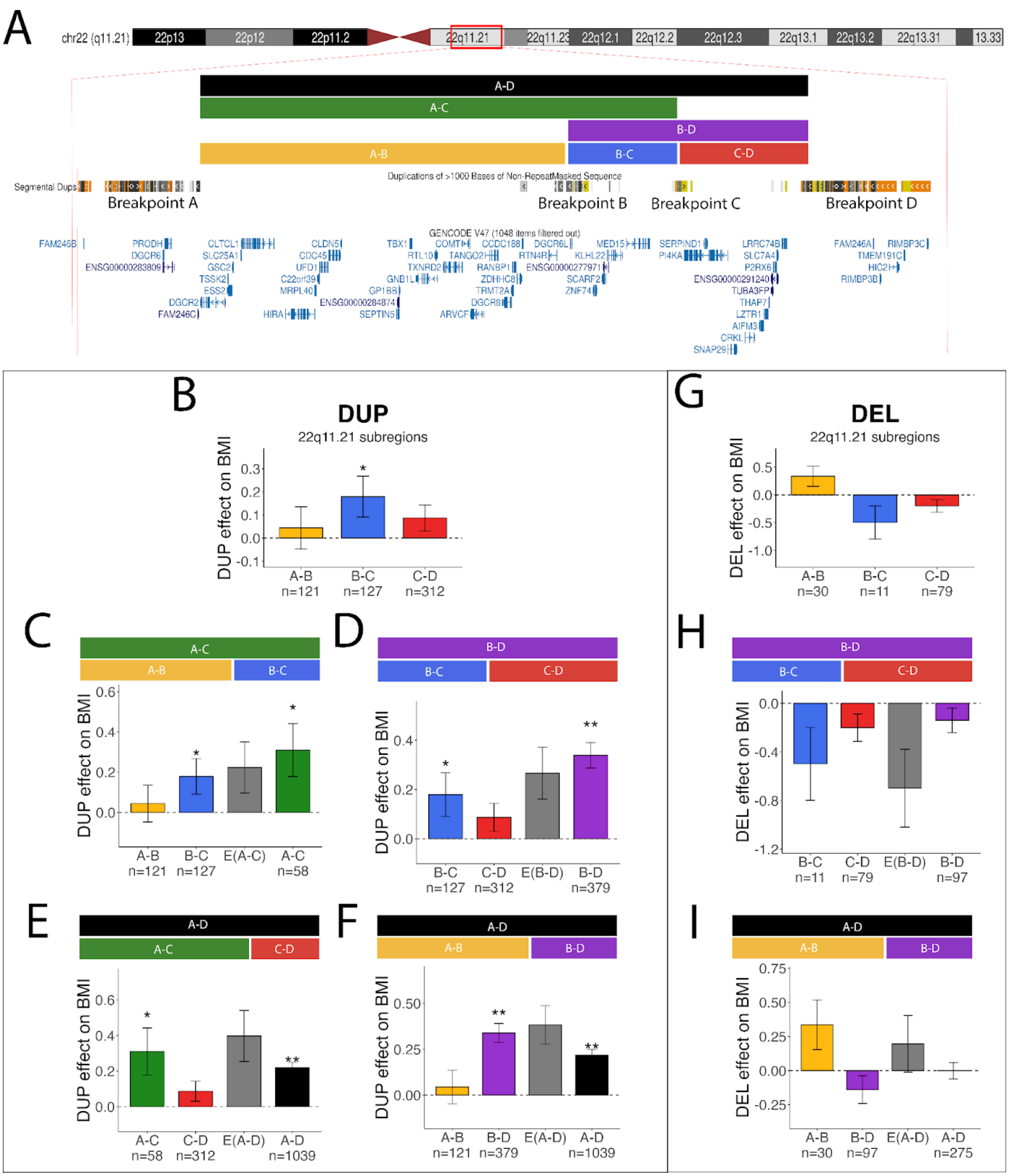

Extended Data Figure 7: Dissection of the 22q11.2 A-D Locus and its effects on BMI. A). Schematic of the genes encompassed by the most commonly observed CNV breakpoints in our study, as defined by Gencode V49 accessed through the UCSC Genome Browser[56] B-C). Breakdown of the effects of DUPs (B-F) and DELs (G-I) of 22q11.2 subregions. E(A-C) denotes the expected value of the effect size of A-C based on the sum of the effects of subregions A-B and B-C. Summary statistics for panels B-I are in Table S3.

medRxiv preprint doi: https://doi.org/10.64898/2026.04.30.26352063; this version posted May 1, 2026. The copyright holder for this preprint (which was not certified by peer review) is the author/funder, who has granted medRxiv a license to display the preprint in perpetuity.
It is made available under a CC-BY 4.0 International license .

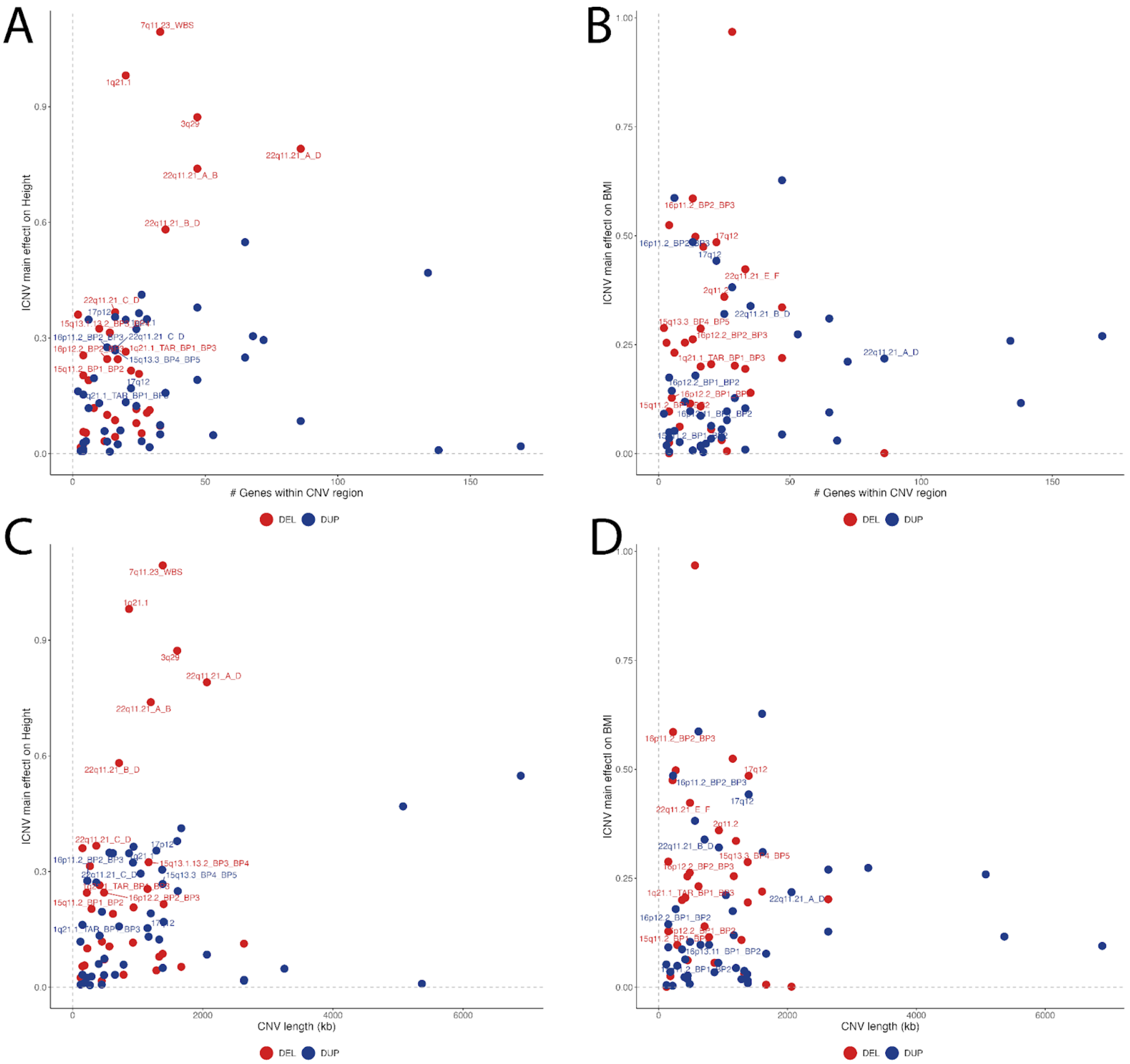

Extended Data Fig. 8: A-B). |CNV effect size| on (A) height and (B) BMI vs. number of genes within CNV region. CNVs with bonferroni significant main effects are labeled. C-D). |CNV effect size| on (C) height and (D) BMI vs. length of CNV region in base-pairs. CNVs with bonferroni significant main effects are labeled

medRxiv preprint doi: https://doi.org/10.64898/2026.04.30.26352063; this version posted May 1, 2026. The copyright holder for this preprint (which was not certified by peer review) is the author/funder, who has granted medRxiv a license to display the preprint in perpetuity.
It is made available under a CC-BY 4.0 International license .

Supplementary Note 1: Model Misspecification

When quantifying genetic or environmental effects on a phenotype with multiple linear regression, there are a few assumptions that must be made in order to assess significance and create confidence intervals for effect size estimates. These assumptions are:

1. Linearity: the response (phenotype) Y is normally distributed about a *linear* combination of the predictor(s) X.
2. Homoscedasticity: the variance of the residuals are equal across X
3. Normality of residuals: the residuals are normally distributed
4. Independence of errors: the y_i are independent from each other… i.e., the phenotype of one sample does not influence the phenotype of another sample
5. Absence of multicollinearity: the predictors X are uncorrelated with each other

When these assumptions are broken, we say that the model is "misspecified", which means that the estimators of the model parameters and their corresponding test statistics do not behave as we would expect them to, which may lead us to incorrectly accept or reject a null hypothesis about the effect of a predictor variable. The effect of model misspecification can vary between parameters and their estimators, and also depends on which assumptions were broken and how they were broken.

There are a few characteristics of genetic and phenotypic data that make genetic studies particularly vulnerable to model misspecification. First, phenotypic data is rarely perfectly normally distributed, which can lead to conflicts with assumptions 2 and 3. [20] discuss the limits of the normal approximation for height, and demonstrate that a log-normal approximation may be more accurate. Second, when studying individuals in a population, it is rare that errors are independent (assumption 4), especially when closely related individuals are involved in the same study. Beyond genetic relationships between individuals, genetic variants are often correlated *within* an individual due to either linkage disequilibrium (LD), population stratification, or assortative mating, which conflicts with assumption 5.

With real world data, it is unlikely that a linear model will be perfectly specified, and it is usually sufficient for the assumptions to be approximately satisfied. However, there is evidence that the effects of model misspecification can be amplified when estimating interaction terms, which will be discussed at length in the following sections. In this work, we are particularly interested in estimating non-additive (or interaction) effects between genetic factors. Thus, it is imperative that we understand the effects of model misspecification on the interaction estimates in this study, and apply the appropriate methods to avoid misspecifying the model in the first place.

Non-normality of the phenotype of interest

A common theme that has arisen in previous work investigating non-additive genetic effects on BMI and height [5,20,83] is that applying the normal approximation to non-normal phenotypes can

medRxiv preprint doi: https://doi.org/10.64898/2026.04.30.26352063; this version posted May 1, 2026. The copyright holder for this preprint (which was not certified by peer review) is the author/funder, who has granted medRxiv a license to display the preprint in perpetuity.
It is made available under a CC-BY 4.0 International license .

result in type 1 errors. Non-normality can often be resolved by applying one of a few transformations to the data. A common transformation is the non-parametric rank based inverse normal transformation (RINT), which ranks the phenotype values and fits their quantiles to a normal distribution. [84] demonstrated that applying this transformation ameliorates the non-normality issue when testing for interactions of common SNPs and age on BMI. However, [85] demonstrated that applying an INT can reduce power to detect epistatic interactions. Due to the already limited power in our study (see Supp Note 2), we decided that a parametric transformation would be more appropriate. In their study of genetic influence within-person longitudinal change in anthropometric traits in UKBB, [5] applied a BoxCox transformation to remove the strong mean-variance relationship in BMI and other traits. We chose this transformation for our study and applied it as in [5] (see methods). All results discussed in the main text use this transformation.

In the context of interactions between rare variants of large effects and PGS, even small deviations from the normal approximation can lead to *biased* estimates of interaction terms, which can lead us to make conclusions about these interactions that are entirely scale dependent. By the central limit theorem, a PGS is normally distributed, as it is the sum of many independent effects. If the phenotype of interest is not normally distributed, a normally distributed PGS *cannot* have a linear relationship with the phenotype while maintaining homoscedasticity, as there is no linear transformation of a normal random variable that is non-normal. This remains true when we introduce a variant of large effect (CNV) to the model… the distribution of the effect of CNV + PGS remains normal when we add the constant CNV effect. However, introducing an interaction term allows the PGS effect to vary depending on CNV background. If the relationship between PGS and the phenotype is non linear, then the variance in the phenotype that can be explained by the PGS will vary across the phenotype distribution. As the magnitude of the CNV effect increases, the effect of the PGS will change with respect to its effect in someone with no CNV, since the variance in phenotype is not constant. Thus, CNVs with large effects will appear to have large interaction effects with the PGS.

This all sounds very theoretical, but if we aren't careful, it can lead us to some very false conclusions, especially in the rare variant space. To illustrate this, we fit two CNVxPGS interaction models for 29 well-powered CNVs in the UKBB for both height and BMI. For height, we used the 1) normally distributed BoxCox transformed phenotype values (which are very similar to the raw height values), and 2) an additional set of phenotype values obtained by squaring the BoxCox values (and keeping the sign). This height distribution has much heavier tails than the normal distribution. For BMI, we used 1) the normally distributed BoxCox transformed BMI values, and 2) the raw BMI values, which have a strong rightward skew. We observed that the main effects of the CNVs were not very sensitive to these transformations. However, the change in interaction effect estimates increased as the magnitude of the CNV main effect increased. For height, the estimates were biased positively, as there is more variance in the tails of the squared height distribution than the normal distribution. This could lead one to conclude that "PGS tends to have a stronger effect in those carrying CNVs with large effects on height". For BMI, the estimates were biased negatively for CNVs that decreased BMI, since there is less variance in the lower end of the raw distribution compared to the normal distribution. The estimates were biased positively for CNVs that increased BMI, since there is

medRxiv preprint doi: https://doi.org/10.64898/2026.04.30.26352063; this version posted May 1, 2026. The copyright holder for this preprint (which was not certified by peer review) is the author/funder, who has granted medRxiv a license to display the preprint in perpetuity.
It is made available under a CC-BY 4.0 International license .

more variance in the upper end of the raw distribution than the normal distribution. This may lead one to conclude that the interactions are "synergistic", where the strength of the PGS as a predictor of BMI changes linearly as a function of rare variant background. These results demonstrate that in the rare variant space, model misspecification resulting from phenotypes with non-normal distribution can lead to conclusions about the nature of genetic interactions that are scale dependent, and that this effect increases as rare variant effect increases.

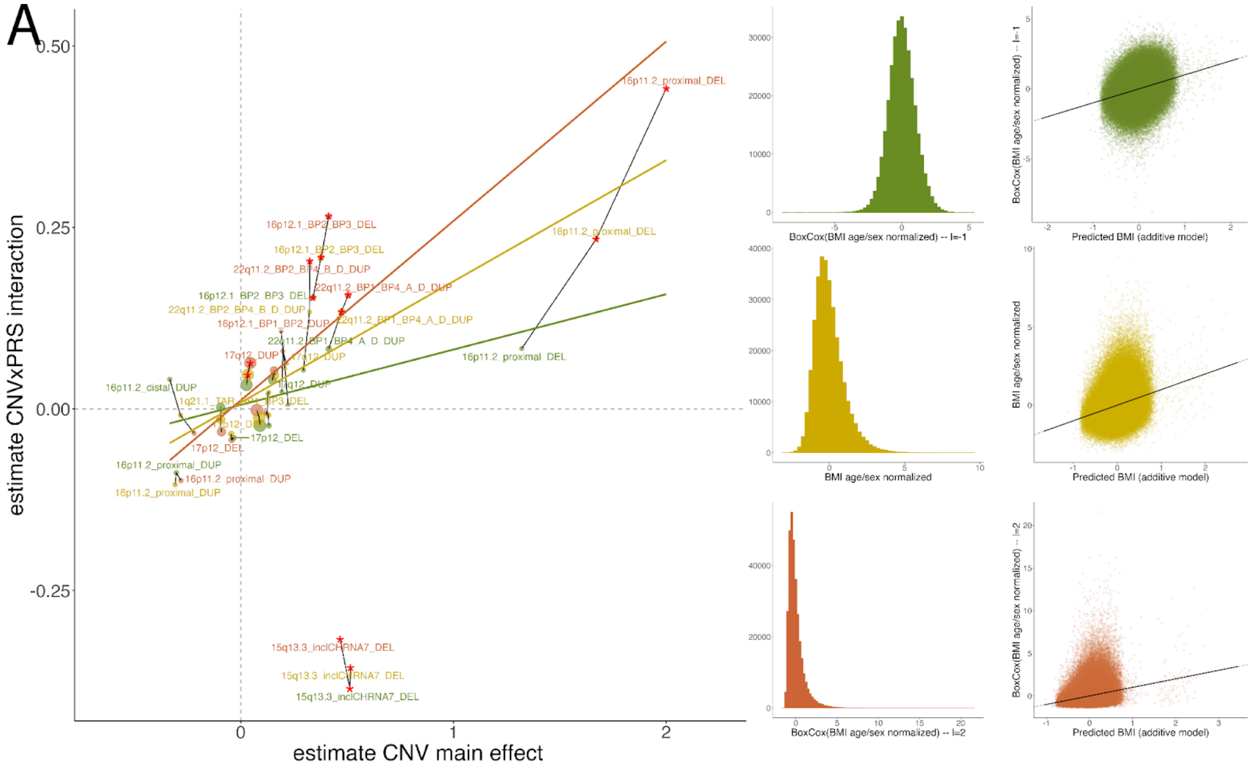

Linkage disequilibrium, population stratification, and assortative mating

A second manner in which genetic studies are especially prone to model misspecification is that genetic variants are often correlated with each other, whether it be on the same chromosome through LD, or across chromosomes through population stratification and assortative mating. It is widely accepted that SNP effect size estimates in a GWAS are inflated due to LD, and this is often a consideration when constructing PGSs from GWAS results (addressed by LD pruning). There is not a clear consensus on how to handle LD in studies of statistical epistasis in the SNP space. One paper conducted a genome wide scan for epistasis between SNPs on a variety of traits, and reported that most of their significant hits were between pairs of SNPs within 1Mb of each other [86]. Other studies removed SNP pairs that are known to be in LD[87]. For the rare variants in our study, which likely arise de Novo, LD with SNPs is not a concern, though they may still be subject to the effects of population stratification or assortative mating if they were inherited. It has been demonstrated that cross chromosome correlations between SNPs (population stratification or assortative mating) can artificially inflate marker-based heritability estimators [88], though there are no studies to our knowledge that address this issue in the context of detecting epistasis. A common practice is to "control" for population stratification by

medRxiv preprint doi: https://doi.org/10.64898/2026.04.30.26352063; this version posted May 1, 2026. The copyright holder for this preprint (which was not certified by peer review) is the author/funder, who has granted medRxiv a license to display the preprint in perpetuity.
It is made available under a CC-BY 4.0 International license .

including ancestry principal components when regressing phenotype on genotype (as we did in this study, see methods), but this does not address the effects of population stratification on estimates of interactions between correlated genetic factors.

Supplementary Note 2: Statistical Power

Even if our model is perfectly specified (see Supp Note 1), there is still the issue of statistical power. Power to detect both main effects and interaction effects was estimated using a Monte Carlo simulation based method. A population of 1 million with *n* CNV carriers (categorical) were generated for each simulation. PGSs and sex were randomly assigned (assuming no assortative mating). Next, a linear model was used to generate phenotypic values based on the specified CNV main effect or interaction term, and normally distributed residual variance depending on the inclusion of PGS in the model (which was assumed to have R^2=0.1), for a total variance of 1 and mean of 0. Finally, main effect or interaction terms were estimated with linear regression. This was repeated 1000 times for each set of parameters, and power was calculated as the proportion of tests that detected the true underlying effect with alpha=0.05.

We are well powered to detect CNV main effects of +/- 0.85 S.D. with 10 CNV carriers. The majority of main effect point estimates in our study were within this range, with a few CNVs having main effects closer to 1 S.D. Thus, we included all CNVs in our main effect estimates that had at least 10 carriers in at least one cohort. Estimates of CNV main effects generated from fewer than 10 carriers in a single cohort were unstable, so those were excluded.

CNV-PGS interactions are expected to be of a much smaller magnitude than main effects. An interaction effect that exceeds the main effect of the PGS (0.297 S.D. for BMI, 0.597 S.D. for height) is unlikely, since that would mean the PGS effect is either doubled or completely negated in CNV carriers. Since the expected interaction term is lower, our power was more limited in this context. Based on our power calculations below, we only included CNVs with over 200 carriers in all cohorts combined in our interaction tests.

For a categorical X categorical interaction where one category is quite common (i.e., sex or medication use), power was slightly greater than categorical X continuous, since we only need to detect a difference in CNV effect between two equally sized groups. However, it is unlikely again that this interaction is larger than the CNV main effect itself (ie, CNV effect is doubled or completely negated in females vs. males). Thus, we elected to use the same 200 carrier cutoff in testing CNVxSex interactions.

medRxiv preprint doi: https://doi.org/10.64898/2026.04.30.26352063; this version posted May 1, 2026. The copyright holder for this preprint (which was not certified by peer review) is the author/funder, who has granted medRxiv a license to display the preprint in perpetuity.
It is made available under a CC-BY 4.0 International license .

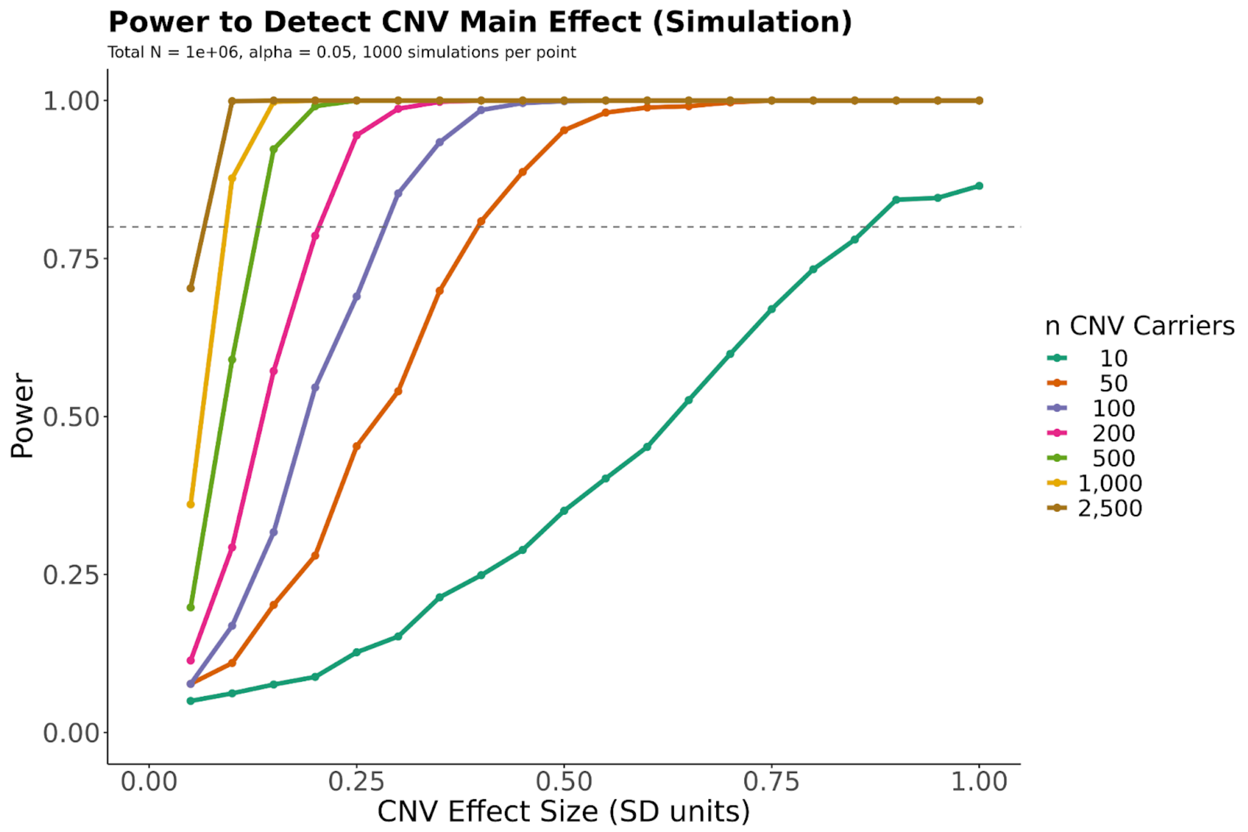

1

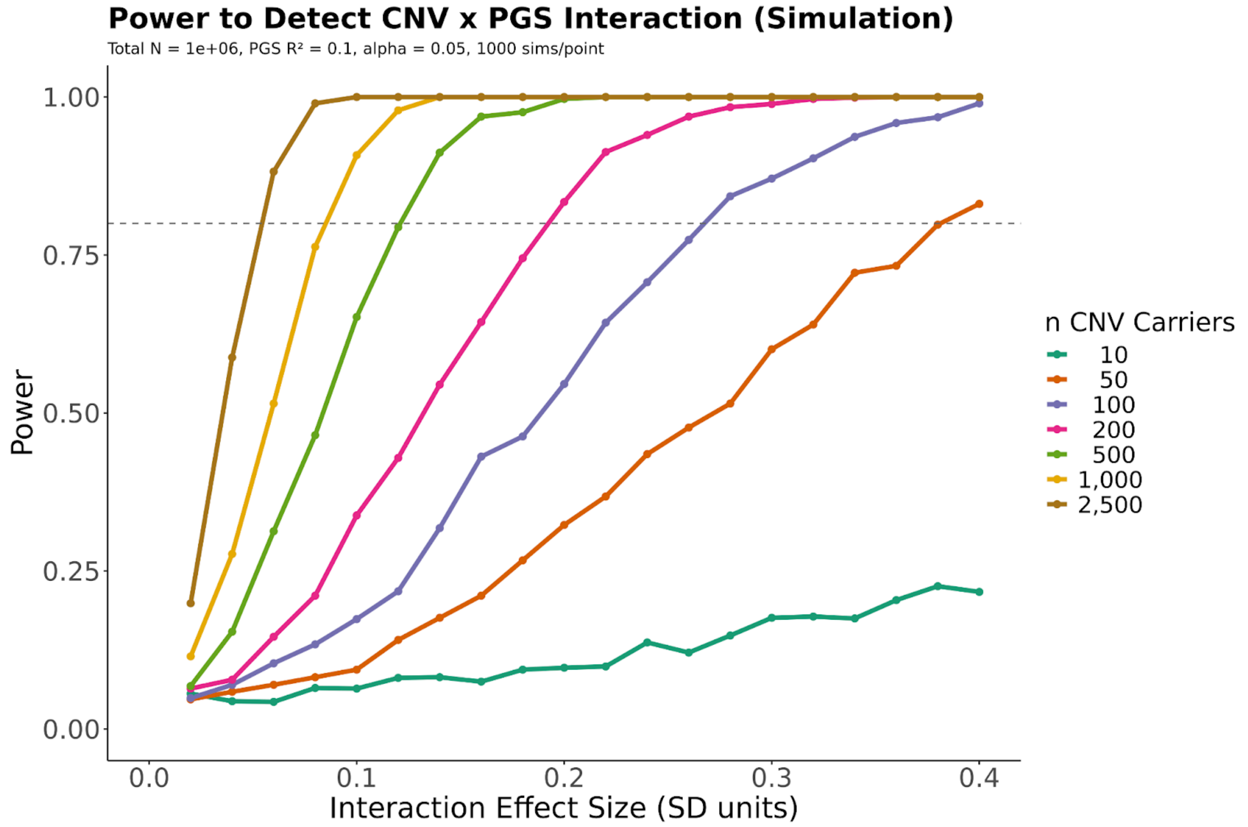

2

medRxiv preprint doi: https://doi.org/10.64898/2026.04.30.26352063; this version posted May 1, 2026. The copyright holder for this preprint (which was not certified by peer review) is the author/funder, who has granted medRxiv a license to display the preprint in perpetuity.
It is made available under a CC-BY 4.0 International license .

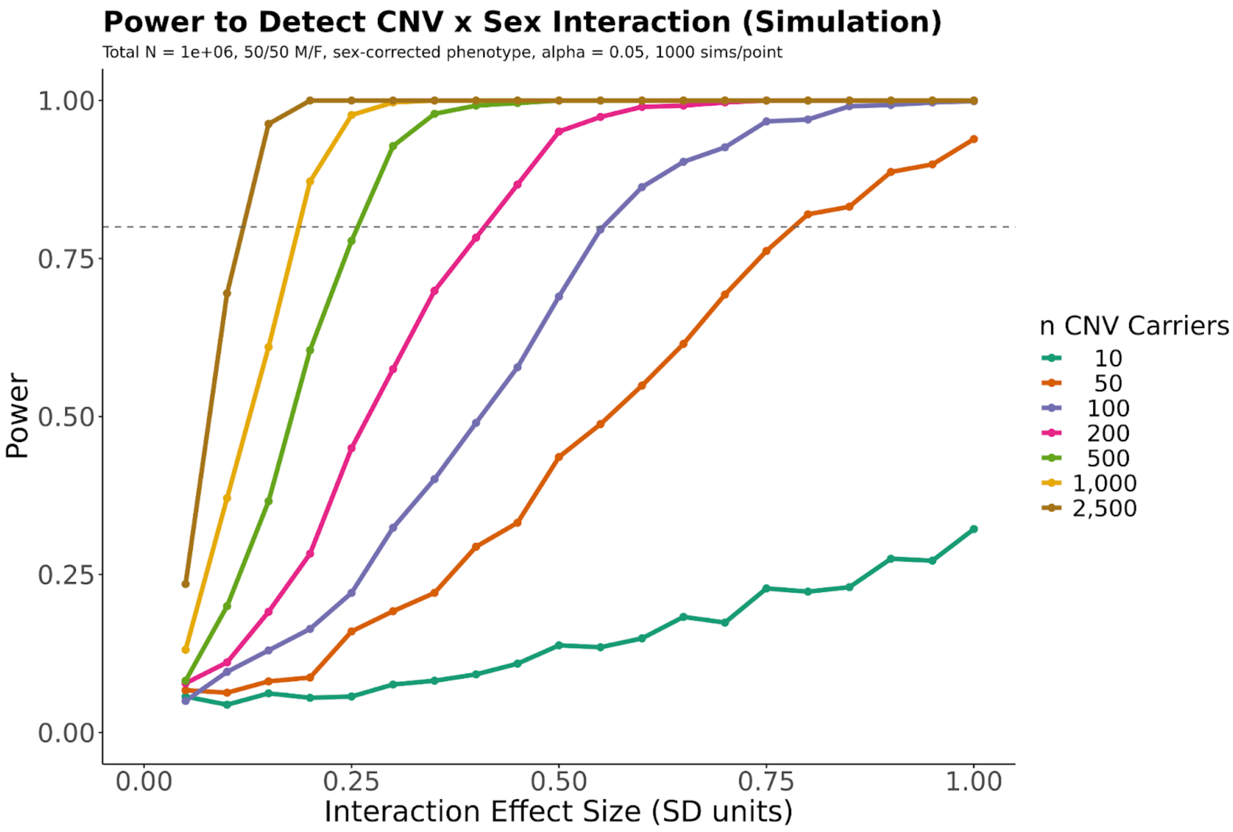

Supplementary Note 3: Comparison of our results with Milind et al.

Theoretical model of dose-response curves by Milind et al.[58] emphasizes that non-monotonic gene-dosage response curves may be a common property of height-associated loci, such that both deletions and duplications typically shift the trait in the same direction (**Millind et al Fig 1F**). Our results show that a subset of loci for height (~30%) exhibit asymmetric effects, and subset of these (e.g. 22q11.2 A-B and 22q11.2 A-D) are non-monotonic such that effects are negative for both DEL and DUP. However, our data suggest that a U-shaped non-monotonic relationship is not an intrinsic property of most loci. Some examples of buffering appear to be explainable by the aggregate behavior of multiple genes within the A-D a locus. Attenuation of the A–D DUP effect is consistent with partial (additive) cancellation of opposing effects from A–B and B–D subregions (**Fig. 6F**). Conversely, when the combined effect of A-B and B-D DELs, both of which are in the negative direction, we see a sub-additive effect (**Fig. 6I**). Thus effects appear to be attenuated in the positive direction for DUP and attenuated in the negative direction for DEL. To summarize, our results are consistent with results from Milind et al in some respects: gene dosage effects on height show evidence of buffering, and effects are biased in the negative direction. Our results contrast in other respects: Non-monotonicity is not a general property of the trait, it is restricted to a specific subset of loci. Buffering appears to involve

medRxiv preprint doi: https://doi.org/10.64898/2026.04.30.26352063; this version posted May 1, 2026. The copyright holder for this preprint (which was not certified by peer review) is the author/funder, who has granted medRxiv a license to display the preprint in perpetuity.
It is made available under a CC-BY 4.0 International license .

combinations of multiple genes, and we see evidence that buffering can stabilize effects in both directions.

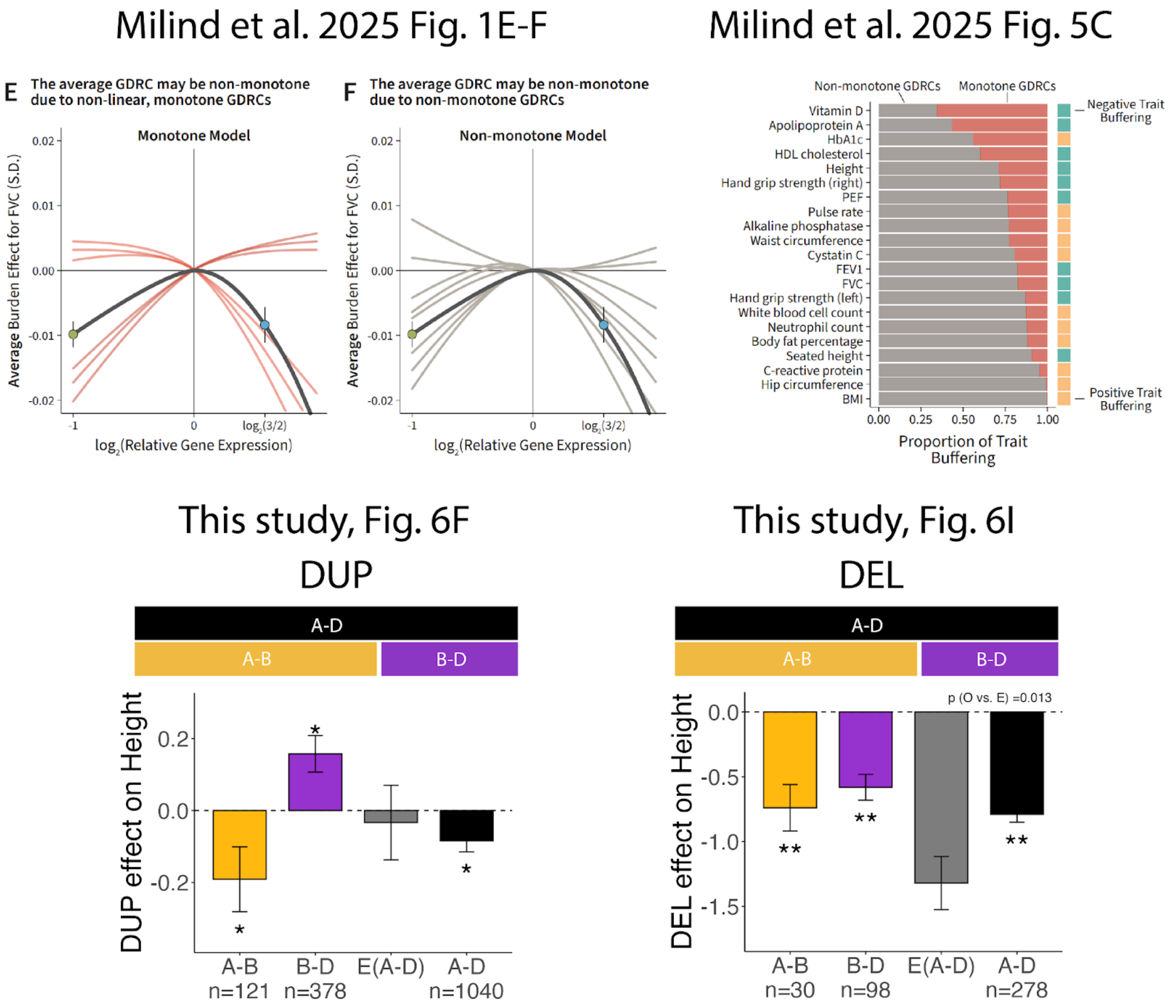

Supplement: 1 [file NIHPP2026.04.30.26352063V1-supplement-1.pdf]
